# Heat stress responses and population genetics of the kelp *Laminaria digitata* (Phaeophyceae) across latitudes reveal differentiation among North Atlantic populations

**DOI:** 10.1002/ece3.6569

**Published:** 2020-08-17

**Authors:** Daniel Liesner, Louise Fouqueau, Myriam Valero, Michael Y. Roleda, Gareth A. Pearson, Kai Bischof, Klaus Valentin, Inka Bartsch

**Affiliations:** ^1^ Alfred Wegener Institute Helmholtz Centre for Polar and Marine Research Bremerhaven Germany; ^2^ UMI EBEA 3614, Evolutionary Biology and Ecology of Algae, CNRS Sorbonne Université, UC, UACH, Station Biologique de Roscoff Roscoff Cedex France; ^3^ Norwegian Institute of Bioeconomy Research Bodø Norway; ^4^ The Marine Science Institute, College of Science University of the Philippines, Diliman Quezon City Philippines; ^5^ Centre for Marine Sciences (CCMAR) University of Algarve Faro Portugal; ^6^ Marine Botany University of Bremen Bremen Germany

**Keywords:** growth rate, local adaptation, marine forest, marine heatwave, microsatellite, physiology

## Abstract

To understand the thermal plasticity of a coastal foundation species across its latitudinal distribution, we assess physiological responses to high temperature stress in the kelp *Laminaria digitata* in combination with population genetic characteristics and relate heat resilience to genetic features and phylogeography. We hypothesize that populations from Arctic and cold‐temperate locations are less heat resilient than populations from warm distributional edges. Using meristems of natural *L. digitata* populations from six locations ranging between Kongsfjorden, Spitsbergen (79°N), and Quiberon, France (47°N), we performed a common‐garden heat stress experiment applying 15°C to 23°C over eight days. We assessed growth, photosynthetic quantum yield, carbon and nitrogen storage, and xanthophyll pigment contents as response traits. Population connectivity and genetic diversity were analyzed with microsatellite markers. Results from the heat stress experiment suggest that the upper temperature limit of *L. digitata* is nearly identical across its distribution range, but subtle differences in growth and stress responses were revealed for three populations from the species’ ecological range margins. Two populations at the species’ warm distribution limit showed higher temperature tolerance compared to other populations in growth at 19°C and recovery from 21°C (Quiberon, France), and photosynthetic quantum yield and xanthophyll pigment responses at 23°C (Helgoland, Germany). In *L. digitata* from the northernmost population (Spitsbergen, Norway), quantum yield indicated the highest heat sensitivity. Microsatellite genotyping revealed all sampled populations to be genetically distinct, with a strong hierarchical structure between southern and northern clades. Genetic diversity was lowest in the isolated population of the North Sea island of Helgoland and highest in Roscoff in the English Channel. All together, these results support the hypothesis of moderate local differentiation across *L. digitata's* European distribution, whereas effects are likely too weak to ameliorate the species’ capacity to withstand ocean warming and marine heatwaves at the southern range edge.

## Graphical abstract

We aimed to understand the intraspecific thermal variation of a coastal foundation species, the kelp *Laminaria digitata*. Marginal populations in particular differed in their response to heat stress treatments, while multilocus genotypes showed a clear and broad separation of *L. digitata* populations into a “northern” and a “southern” clade. These results imply that *L. digitata* populations have phenotypically diverged under the influence of the Quaternary glacial cycles.

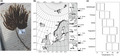

## 1 INTRODUCTION

Temperature is one of the main drivers determining latitudinal species distributions on the global scale (Jeffree & Jeffree, 1994; Lüning, 1990; Stuart‐Smith, Edgar, & Bates, 2017). For sedentary organisms, the thermal limits of the realized niche are broadly described by mean summer and winter isotherms (van den Hoek, 1982; Jeffree & Jeffree, 1994; Stuart‐Smith et al., 2017), between which a species can complete its life cycle, while single extreme temperature events can further alter local species abundances especially at the range edges (Ruthrof et al., 2018; Smale, Wernberg, & Vanderklift, 2017; Straub et al., 2019). As a result of climate change, isotherms in the northern hemisphere have been shifting predominantly poleward since 1960 (Burrows et al., 2011), with consequent phenological and distributional changes in many taxa (Chen, Hill, Ohlemüller, Roy, & Thomas, 2011; Poloczanska et al., 2013).

Predictions of species distributions during climate change are often based on niche models, which assume that all individuals within a species respond uniformly (King, McKeown, Smale, & Moore, 2018; Müller, Laepple, Bartsch, & Wiencke, 2009; Reed, Schindler, & Waples, 2011). Consequently, trait variability needs to be integrated into estimates of future range shifts (Bennett, Duarte, Marbà, & Wernberg, 2019; Cacciapaglia & van Woesik, 2018; Chardon, Pironon, Peterson, & Doak, 2020), especially as recent evidence suggests a central role of plasticity and local adaptation in species’ responses to climate change (Atkins & Travis, 2010; Liesner, Shama, Diehl, Valentin, & Bartsch, 2020; Valladares et al., 2014).

Along cold‐temperate to polar rocky shores, kelps (large brown algae in the order Laminariales) provide important ecosystem services as foundation species of marine forests (Steneck et al., 2002; Teagle, Hawkins, Moore, & Smale, 2017; Wernberg & Filbee‐Dexter, 2019). Their coastal habitats are highly affected not only by gradual global warming, but also further by the accompanying changing onset of the warm season (Lima & Wethey, 2012) as well as the frequency and magnitude of extreme temperature events such as marine heatwaves (MHW; Hobday et al., 2016; Oliver et al., 2018). Poleward range shifts have already been documented for various kelp and fucoid seaweeds, which were attributed to global warming (Lima, Ribeiro, Queiroz, Hawkins, & Santos, 2007; Nicastro et al., 2013; Smale, Wernberg, Yunnie, & Vance, 2015).

Further range shifts are predicted for many species, including the North Atlantic kelp *Laminaria digitata* (Hudson) J.V. Lamouroux (Assis, Araújo, & Serrão, 2018; Raybaud et al., 2013). At high latitudes, *L. digitata* occurs on Spitsbergen and Greenland, while its southern distribution limit along the European coastline is in Brittany, France (Lüning, 1990). It thereby occurs between the 0°C winter and 18°C summer sea‐surface isotherm (Müller et al., 2009) indicating its wide temperature performance range as an Arctic to cold‐temperate species (sensu Lüning, 1990). Comparative laboratory studies described an upper survival temperature of western and eastern Atlantic juvenile *L. digitata* sporophytes of 23°C over seven days (Bolton & Lüning, 1982) and of 21°C over 14 days (tom Dieck, 1992), indicating high stability of thermal characteristics across regions. However, these investigations only compared single unialgal strains, which may not represent the entire species. Investigations on wild *L. digitata* sporophytes from Nova Scotia show mortality within one week at 21°C and tissue damage at 18°C (Simonson, Scheibling, & Metaxas, 2015). In South West England *L. digitata*, stress signals and reduced growth were evident after 16 days at 18°C (Hargrave, Foggo, Pessarrodona, & Smale, 2017).

*L. digitata* is a relatively young species, which probably originated from a Pacific ancestor crossing the Arctic toward the Atlantic ca. 5.3 million years ago (Lüning & tom Dieck, 1990; Rothman, Mattio, Anderson, & Bolton, 2017; Starko et al., 2019). Therefore, *L. digitata* was likely present in the Atlantic over multiple glacial cycles during the Quaternary (Assis et al., 2018), including the most recent Last Glacial Maximum 20,000 years ago (LGM; Clark et al., 2009). Recently, it has been proposed that *L. digitata* persisted during the LGM in only two disjoint refugia in the Northeast Atlantic, one located in the Armorican/Celtic Sea and one further north in the region of Ireland and Scotland (Neiva et al., 2020). Such a northern refugium for *L. digitata* was also suggested by King et al. (2020). Therefore, not only might the current climate since the LGM have affected thermal plasticity of *L. digitata* populations, but also the repeated retreat into glacial refugia and subsequent recolonization of the Northern Atlantic might have modulated genetic diversity and structure over several glacial cycles (Hewitt, 2004; Maggs et al., 2008). This possibly facilitated phenotypic divergence along what is presently a widespread latitudinal distribution gradient.

Local adaptation can occur along environmental gradients or in populations under unique selection pressures and affects response traits to increase the fitness of individuals in their specific environment (Kawecki & Ebert, 2004). For populations at their ecological range margins (i.e., marginal populations sensu Soulé, 1973), the unfavorable local environment can result in smaller population size and low genetic diversity (Eckert, Samis, & Lougheed, 2008; Hampe & Petit, 2005; Kawecki, 2000). Therefore, genetic drift may impair natural selection leading to maladaptation in marginal populations (Eckert et al., 2008; Pearson, Lago‐Leston, & Mota, 2009). Conversely, a highly selective environment at a species’ range margin might eventually facilitate local adaptation in these unique populations (reviewed by Hardie & Hutchings, 2010) and even increase their performance following climate change (Halbritter, Billeter, Edwards, & Alexander, 2015).

Meanwhile, there is much evidence for intraspecific variation among populations of seaweeds and seagrass (reviewed by King, McKeown, et al., 2018). Local adaptation might be common in kelps and seaweed populations generally, due to their low dispersal capacity and strong spatial structuring (King, McKeown, et al., 2018; Miller et al., 2019). Studies on local adaptation in *L. digitata* suggest that differentiation between populations could have occurred due to their geographic position (range central and marginal as well as southern and northern). King et al. (2019) investigated the expression of genes coding for heat shock proteins (HSP) in response to an hour‐long heat shock in *L. digitata* from Scotland (range center) and Southern England (trailing edge). Maximum HSP response was present at 4–8°C higher temperatures in the southern populations in this short‐term study, despite comparably low genetic diversity (King et al., 2020). The reduced genetic diversity and altered reproductive strategy in a southern marginal population in Brittany, France, also suggests that local differentiation has taken place (Oppliger et al., 2014; Valero et al., 2011). Overall, research on integrative responses such as growth is lacking when assessing the intraspecific thermal variation of *L. digitata*. Additionally, few studies on thermal responses of kelps incorporate physiology and population genetics over large geographic scales, although they may help to better predict climate change effects (Nepper‐Davidsen, Andersen, & Pedersen, 2019).

The main objective of this study was thus to assess differentiation in heat stress responses among populations of *Laminaria digitata* present along the entire Northeast Atlantic distribution zone through a mechanistic, common‐garden experiment. We hypothesized that an increasing thermal selection pressure toward the southern distribution limit increased heat resilience of sporophytes from southern *L. digitata* populations. Because of high similarities of thermal characteristics across regions reported in previous comparative studies (Bolton & Lüning, 1982; tom Dieck, 1992), we expected local differentiation in response to heat to be of small extent and to occur mainly toward the upper temperature limit (see also King et al., 2019). We further expected phenotypic differentiation to occur more prominently in populations experiencing low amounts of gene flow, while we expected low genetic diversity to be associated with reduced heat resilience as a result of genetic drift and possible maladaptation, which we investigated by the use of neutral microsatellite markers.

## 2 MATERIAL AND METHODS

### 2.1 Sample collection and preparation

**Figure 1:**
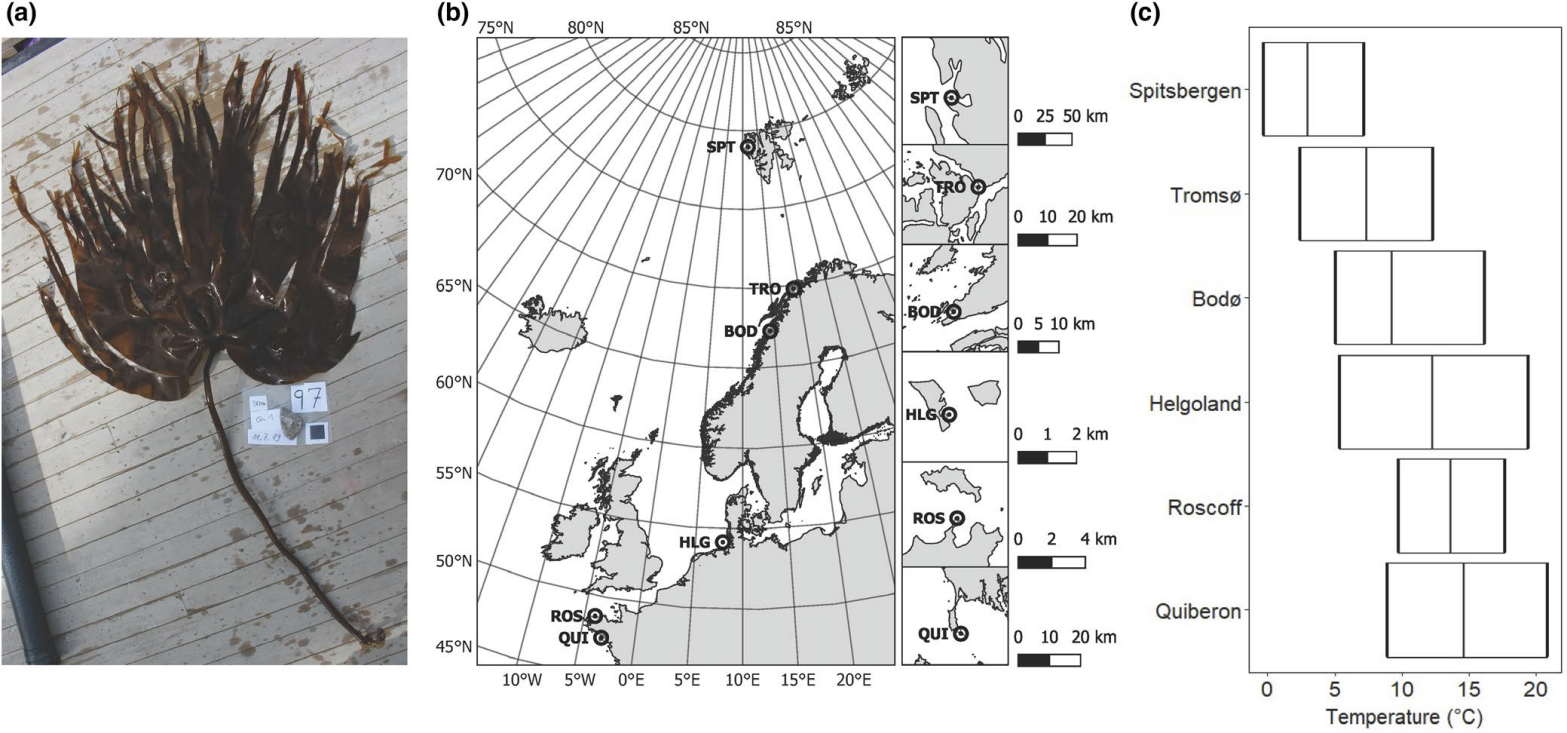
(a) Seven‐year‐old *Laminaria digitata* sporophyte from Spitsbergen, July 2019. The black reference square measures 5 × 5 cm. (b) Sampling locations of the *L. digitata* populations used in this study and (c) temperature amplitudes in 2018 marking minimum, mean, and maximum temperatures based on satellite‐obtained mean daily sea‐surface temperature datasets (E.U. Copernicus Marine Service, 2019). Abbreviations: BOD, Bodø; HLG, Helgoland; QUI, Quiberon; ROS, Roscoff; SPT, Spitsbergen; TRO, Tromsø

We collected 30–35 fertile *L. digitata* sporophytes (Figure 1a) from the low intertidal zone, ensuring a distance of >1 m between samples (for the samples collected by diving in Spitsbergen, this was not guaranteed), in each of the following locations during summer (Figure 1b): Stuphallet, Kongsfjorden, Spitsbergen, Norway (SPT; 78.975 N, 11.633 E; 16 July 2019; approximate SST at time of sampling: 6.5°C); north of Tromsø, Norway (TRO; 69.790 N, 19.054 E; 14 August 2018; 8.5°C); Bodø, Norway (BOD; 67.284 N, 14.383 E; 12 June 2018; 9°C); Helgoland, Germany (HLG; 54.178 N, 7.893 E; 13 August 2018; 18°C); Roscoff, France (ROS; 48.727 N, 4.005 W; 11 September 2018; 16.5°C); and Quiberon, France (QUI; 47.470 N, 3.091 W; 10 September 2018; 16°C). Sampling in Norway and France and handling of data was conducted in accordance with the French legislation on the Access to Genetic Resources and Benefit‐Sharing. Maps (Figure 1b) were generated using a European Environment Agency coastline shapefile (European Environment Agency, 2019) and QGIS 3.8.2‐Zanzibar software (QGIS Development Team, 2019). To represent the current temperature ranges experienced by the sampled sporophytes, satellite‐obtained daily mean sea‐surface temperature data (Figure 1c) with a resolution of 0.05° × 0.05° were generated representatively for 2018 using E.U. Copernicus Marine Service Information (E.U. Copernicus Marine Service, 2019).

Entire sporophytes were stored in ambient seawater for up to two days before processing. At the sampling locations, clean material from the meristematic region was preserved in silica gel for microsatellite genotyping. For the heat stress experiment, six disks (Ø 20 mm) were cut from the meristematic region of each sporophyte (i.e., 180 disks per population) in a distance of 5–10 cm from the stipe‐blade transition zone. Disks were stored moist in cool boxes (<15°C) and transported to the laboratory within 30 hr. All experiments were performed at the Alfred Wegener Institute in Bremerhaven, Germany.

### 2.2 Heat stress experiment

#### 2.2.1 Experimental design

**Figure 2:**
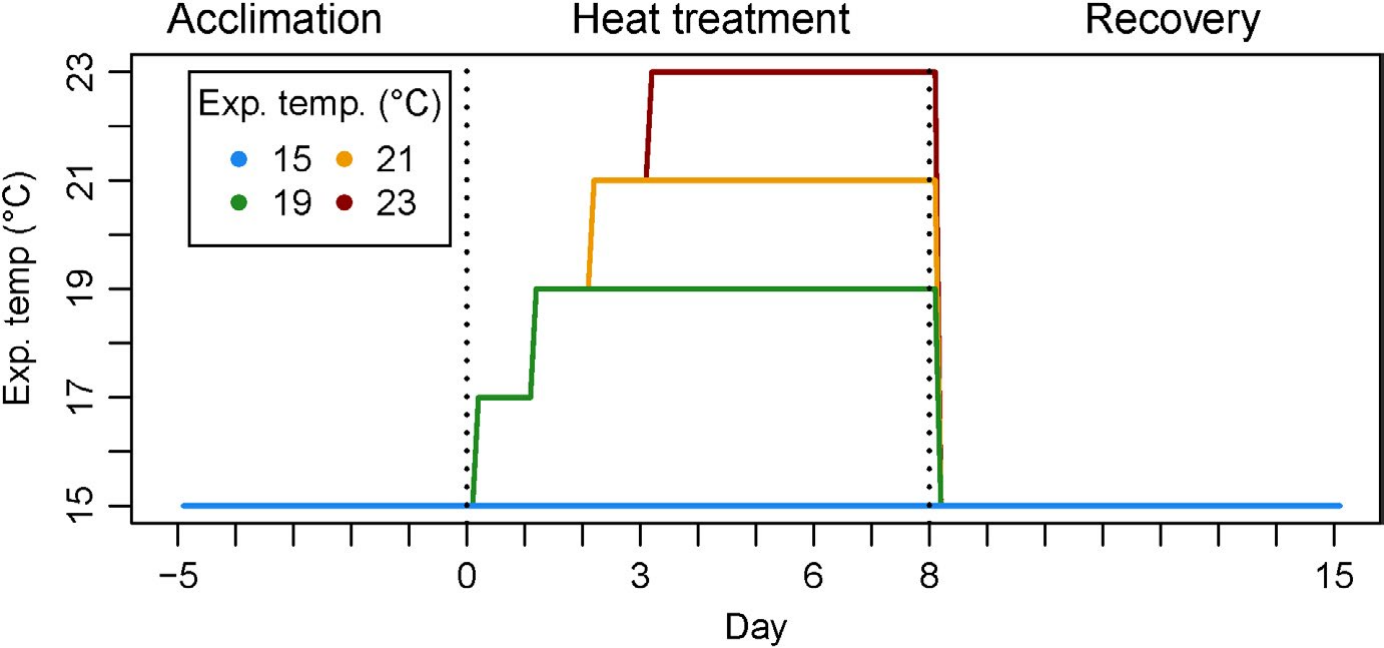
Timeline of the heat stress experiment of *Laminaria digitata*. Dotted lines separate experimental phases of acclimation at 15°C (days −5–0), heat treatment (days 0–8), and recovery at 15°C (days 8–15). Growth and F_v_/F_m_ were measured on days −5, 0, 3, 6, 8, and 15. On days 0 and 8, rapid light curves were performed and samples were frozen for biochemical and pigment analyses

We designed the experiment (Figure 2) as a mechanistic short‐term exposure to heat stress around the upper survival temperature of *L. digitata* sporophytes (21°C for a two week exposure; tom Dieck, 1992). A temperature of 19°C was considered to be a sublethal treatment for all populations, 21°C a threshold treatment (lethal over a longer exposure time; tom Dieck, 1992; Wilson, Kay, Schmidt, & Lotze, 2015), and 23°C a critical stress treatment (Bolton & Lüning, 1982), which also surpassed mean daily maximum temperatures of all sampled populations in 2018 (Figure 1c). We exposed all samples to the same temperatures, irrespective of the ecological significance for local populations, to investigate the thermal plasticity and potential of *L. digitata* across its entire distribution range. The heat stress experiment was conducted in independent runs in common‐garden conditions with material from Spitsbergen, Tromsø, Helgoland, Roscoff, and Quiberon. Due to logistic constraints, Bodø had to be excluded, and Spitsbergen material was only tested for growth and fluorescence characteristics and not for biochemistry and pigments.

For each population, five replicate pools each contained all meristem disks of six distinct sporophytes to prevent pseudoreplication. Meristem disks were transferred into sterile 5 L glass bottles filled with modified half‐strength Provasoli‐enriched natural seawater (PES; Provasoli, 1968; modifications: HEPES buffer instead of TRIS, double concentration of Na_2_glycerophosphate; iodine enrichment following Tatewaki, 1966), which was exchanged every 3–4 days. Irradiance ranged between 30 and 35 µmol photons m^−2^ s^−1^ at the bottom of the beakers in a 16:8‐hr light:dark (L:D) cycle (ProfiLux 3 with LED Mitras daylight 150, GHL Advanced Technology, Kaiserslautern, Germany). Beakers were aerated gently to ensure motion of disks and even light and nutrient availability.

To allow recovery from sampling stress, disks were cultivated at 10°C for two (Tromsø) or nine days (Spitsbergen due to logistic issues), or at 15°C for four (Roscoff, Quiberon) or three days (Helgoland) before the acclimation phase of the experiment. From each replicate pool, eight disks were then randomly assigned to one replicate 2 L glass beaker in each of the four temperature treatment groups (15, 19, 21, 23°C, *n = *5). Six disks per replicate were marked by punching a small hole on the outer rim with a Pasteur pipette to be frozen for biochemical and pigment analysis during the experiment. The two unmarked disks were used for growth and fluorometric measurements over the course of the experiment.

At the beginning of the experiment, disks were acclimated at 15°C for five days to obtain a similar metabolic state (day −5 to day 0; Figure 2). Although the northern populations Spitsbergen and Tromsø do not usually experience temperatures this high (Figure 1c), 15°C is a temperature within the growth optimum of *L. digitata* (Bolton & Lüning, 1982; tom Dieck, 1992), which is considered to be stable (Wiencke, Bartsch, Bischoff, Peters, & Breeman, 1994), even for the Spitsbergen population (Franke, 2019). Starting the heat stress treatment on day 0, temperature was increased by increments of 2°C day^−1^ until the desired temperature was reached. The maximum temperature 23°C was applied for five days, while 21°C and 19°C were applied for six and seven days, respectively, according to the acclimation scheme (Figure 2). On day 8, temperature was set to 15°C for all treatment groups to initiate a recovery period of seven days. Measurements took place at the beginning of the experiment (day −5; Figure 2), the beginning of the heat treatment (day 0), before applying the maximum temperature 23°C (day 3), in the middle of the heat treatment (day 6), at the end of the heat treatment (day 8), and after the recovery period (day 15).

#### 2.2.2 Relative growth rates

Two disks per replicate were repeatedly measured for growth over the course of the experiment (*n = *5). Disks were blotted dry and weighed for growth analyses. Relative growth rates (RGR) were calculated as $\text{RGR}\left({\text{g g}}^{-1}\;{\text{day}}^{-1}\right)=\frac{\ln x_{2}-\ln x_{1}}{t_{2}-t_{1}}$

where *x*_1_ = weight (g) at time 1, *x*_2_ = weight at time 2, *t*_1_ = time 1 in days, and *t*_2_ = time 2 in days.

#### 2.2.3 PAM Fluorometry

Fluorescence parameters were assessed to estimate photoacclimation reactions in response to temperature (Davison, Greene, & Podolak, 1991; Machalek, Davison, & Falkowski, 1996) and were all conducted using a PAM‐2100 chlorophyll fluorometer (Walz, Effeltrich, Germany). Maximum quantum yield of photosystem II (F_v_/F_m_) was repeatedly measured in two disks per replicate over the course of the experiment following 5 min dark acclimation (*n = *5). Before and after the heat treatment (day 0 and day 8), rapid light curves (RLC) were conducted after F_v_/F_m_ measurements on one disk (*n = *3). RLC irradiance steps ranged from 0 to 511 µmol photons m^−2^ s^−1^. Based on the photon flux density (PFD) and the effective quantum yield, relative electron transport rates (rETR) in photosystem II were calculated following Hanelt (2018) as

rETR=PFD×Yield

rETR was plotted against PFD, and the resulting curves were fitted following the model of Jassby and Platt (1976) to calculate the maximum relative electron transport rate rETR_max_, the saturation irradiance I_k_, and the photosynthetic efficiency α of each curve.

Nonphotochemical quenching was calculated following Serôdio and Lavaud (2011) as

$$\text{NPQ}=\frac{F_{m}-Fm^{\text{'}}}{Fm^{\text{'}}}$$

where *F_m_* = maximum fluorescence of a dark‐adapted sample, and *F_m_*′ = maximum fluorescence of a light‐adapted sample.

NPQ versus irradiance curves were fitted following the model of Serôdio and Lavaud (2011) to calculate maximum nonphotochemical quenching NPQ_max_, the saturation irradiance E_50_, and the sigmoidicity coefficient *n*.

#### 2.2.4 Biochemistry

Biochemical and pigment analyses were conducted with material from Tromsø, Helgoland, Roscoff, and Quiberon. We assessed the early photosynthetic product mannitol, which is accumulated during summer (Schiener, Black, Stanley, & Green, 2015), and elemental carbon and nitrogen to estimate carbon assimilation and nutrient storage in response to temperature. In wild sporophytes, assimilated mannitol is metabolized into the long‐term storage polysaccharide laminarin and translocated into the distal thallus (Gómez & Huovinen, 2012; Yamaguchi, Ikawa, & Nisizawa, 1966). As the meristematic region only contains minimal amounts of laminarin in wild sporophytes (Black, 1954), and as maximum laminarin contents occur with a seasonal delay of 1–2 months in late autumn (Haug & Jensen, 1954; Schiener et al., 2015), we did not assess laminarin storage in our short‐term experiment on isolated meristematic disks.

Before the start and at the end of the heat treatment (day 0 and day 8), three disks per replicate beaker (*n = *5) were frozen in liquid nitrogen for biochemical and pigment analysis and stored at −80°C. For mannitol, carbon, and nitrogen analyses, samples were lyophilized and ground to a fine powder. For the analysis of carbon and nitrogen contents, 2–3 mg ground tissue per sample was packed into tin cartridges, compressed, and combusted at 1,000°C in an elemental analyzer (EURO EA, HEKAtech GmbH) with acetanilide as standard. Mannitol was extracted in 70% ethanol from three technical replicates of each experimental sample (Karsten, Thomas, Weykam, Daniel, & Kirst, 1991). Analysis was performed in an HPLC Agilent Technologies system (1200 Series) with an Aminex Fast Carbohydrate Analysis Column HPAP (100 × 7.8 mm, 9 µm, Bio‐Rad, Munich, Germany) protected by a guard cartridge (Phenomenex, Carbo‐Pb‐2 + 4 × 3.00 mm I.D., Aschaffenburg, Germany).

#### 2.2.5 Pigments

We assessed chlorophyll and xanthophyll pigments in response to heat stress as a further indicator of photoprotection (Bischof & Rautenberger, 2012; Uhrmacher, Hanelt, & Nultsch, 1995). Pigment samples were lyophilized separately from biochemical samples (*n = *5). They were ground under dim light conditions, weighed to 50–80 mg, and extracted in 90% aqueous acetone in darkness for 24 hr at 7°C. HPLC analysis followed the protocol and equipment described by Scheschonk et al. (2019), using a LaChromElite system (L‐2200 autosampler with Cooling Unit; DAD detector L‐2450; VWR‐Hitachi International) with a Spherisorb ODS‐2 column (25 cm × 4.6 mm, 5 µm particle size, Waters, Milford, USA) protected by a guard cartridge (LiChrospher 100‐RP‐18; Merck). The elution gradient was applied according to Wright et al. (1991). We used standards of chlorophyll *a* and *c*, fucoxanthin, β‐carotene, violaxanthin, antheraxanthin, and zeaxanthin (DHI lab products, Hørsholm, Denmark). To assess parameters of photoprotection as a stress response, we calculated the mass ratio of xanthophyll pigments violaxanthin (V), antheraxanthin (A), and zeaxanthin (Z) per chlorophyll *a* (Chl *a*) following Bollen, Pilditch, Battershill, and Bischof (2016) as.

$$\text{VAZ}:\text{Chl}\;a\;\text{ratio}\left({\text{mg mg}}^{-1}\text{Chl}\;a\right)=\frac{V+A+Z}{\text{Chl}\;a}$$

and de‐epoxidation ratio of xanthophyll cycle pigments following Colombo‐Pallotta, García‐Mendoza, and Ladah (2006) as.

$$\text{De}-\text{epoxidation ratio}=\frac{Z+0.5A}{V+A+Z}$$

#### 2.2.6 Statistical analyses of physiological parameters

As we measured two disks per replicate, we calculated growth rates and F_v_/F_m_ from mean values per replicate. One disk was removed from the Spitsbergen 23°C treatment due to bleaching during the heating ramp. Despite identification efforts in the field, almost none of the microsatellite markers amplified in two samples from Spitsbergen (see also 2.3.2). This led to the assumption that the two samples were of *Hedophyllum nigripes* (J. Agardh) Starko, S.C.Lindstrom & Martone, which is morphologically very similar to *L. digitata* (Dankworth, Heinrich, Fredriksen, & Bartsch, 2020; Longtin & Saunders, 2015). One replicate pool probably containing meristem disks from both species was therefore removed from the experiment. Due to the mannitol extraction performed in triplicates, means of the three subsamples of each mannitol replicate were analyzed. In carbon and nitrogen analyses, four data points were deleted due to a measuring error on day 0. In the xanthophyll pool and de‐epoxidation analyses, one outlier was deleted due to implausibly high zeaxanthin contents about four times higher than the next highest value.

All analyses of the heat stress experiment were performed in the R statistical environment version 3.6.0 (R Core Team, 2019). We fitted generalized least squares models for all parameters and tested for significance using analyses of variance (ANOVA). All models were fitted using the “gls” function from the R package “nlme” (Pinheiro, Bates, DebRoy, & Sarkar, 2019) with weights arguments to counteract heterogeneity of variance of normalized model residuals (Zuur, Ieno, Walker, Saveliev, & Smith, 2009). Normalized model residuals were assessed with Shapiro–Wilk normality tests and Levene's tests for homogeneity of variance. For repeated measures analyses of variance (RM ANOVA) of growth rates and F_v_/F_m_, temperature, population, and time were modeled as interactive fixed effects and a compound symmetry correlation structure was incorporated using a time covariate and replicate as grouping factor (Pekár & Brabec, 2016; Zuur et al., 2009). Analyses of variance were then performed on the models with the “anova” function to assess the effects of the fixed effects temperature, population and exposure time, and their interactions. For all biochemical, pigment, and fluorometric analyses, initial contents at day 0 were incorporated in the models as covariates to account for baseline differences, and temperature and population were modeled as fixed effects. Analyses of variance were performed to assess the effects of the initial value covariate and the fixed effects temperature and population, and their interaction. Pairwise comparisons were performed using the R package “emmeans” (Lenth, 2019) and using the “Satterthwaite” mode for calculation of degrees of freedom and Tukey adjustment of *p*‐values for multiple comparisons between independent groups. For pairwise comparisons in the repeated measures analyses (growth and F_v_/F_m_), the “df.error” mode for calculation of degrees of freedom was applied. Because of the repeated measures design and because the “df.error” mode overestimates the degrees of freedom (Lenth, 2019), *p*‐values were adjusted by means of the conservative Bonferroni correction for multiple testing to reduce the probability of type I errors. Correlation analyses (Kendall's rank correlation) were conducted between all parameters measured after the heat treatment (relative growth rates calculated between day 0 and day 8) using the “cor.test” function from the default R package “stats” (R Core Team, 2019).

### 2.3 Microsatellite genotyping

#### 2.3.1 DNA extraction

DNA was extracted from 8–12 mg of dried tissue using the NucleoSpin 96 Plant II kit (Macherey‐Nagel GmbH & Co. KG) following the manufacturer's instructions. The lysis, microsatellite amplification and scoring was performed for 12 polymorphic loci following Robuchon, Le Gall, Mauger, and Valero (2014). Multiplex PCRs were modified using 5X GoTaq Flexi colorless reaction buffer (Promega Corp., Madison, USA) instead of 1X and performed using a T100™ Thermal Cycler (Bio‐Rad Laboratories Inc.).

#### 2.3.2 Microsatellite amplification, scoring, and correction

Among the markers used, six were previously developed for *Laminaria digitata* (Ld148, Ld158, Ld167, Ld371, Ld531, and Ld704; Billot et al., 1998) and six for *Laminaria ochroleuca* (Lo4‐24, Lo454‐17, Lo454‐23, Lo454‐24, Lo454‐27, and Lo454‐28; Coelho, Serrão, & Alberto, 2014). Alleles were sized using the SM594 size standard (Mauger, Couceiro, & Valero, 2012) and scored manually using GeneMapper 4.0 (Applied Biosystems). Individuals, for which more than one locus did not amplify, were removed from the dataset. Amplification was faulty for the population of Helgoland sampled in 2018, which could be linked to poor preservation or insufficient dehydration. Therefore, the dataset of the same population sampled at the same site in 2016 was used in the genetic analysis instead. In total, 190 individuals were initially genotyped for twelve microsatellite markers and 179 were retained.

#### 2.3.3 Genetic diversity

Prior to genetic analysis, the presence of null alleles was tested using the ENA method in FreeNa (Chapuis & Estoup, 2007). Single and multilocus estimates of genetic diversity were calculated for each population as the mean number of alleles per locus (N_a_), unbiased expected heterozygosity (H_e_, sensu Nei, 1978), observed heterozygosity (H_o_), and number of private alleles (P_a_) using GenAlEx 6.5 (Peakall & Smouse, 2006). In addition, allelic richness (AR) was computed using FSTAT 2.9.3 (Goudet, 2001) for each locus using the rarefaction method. Linkage disequilibrium between pairs of loci and single estimates of deviation from random mating (F_IS_) was calculated according to Weir and Cockerham (1984), and statistical significance was computed using FSTAT based on 7920 permutations for linkage disequilibrium and 10^4^ for F_IS_. To test the null hypothesis that populations did not differ in genetic diversity, a one‐way ANOVA was performed for AR, P_a,_ and H_e_ in R (R Core Team, 2019). Pairwise differences between means were tested by Fisher Individual Tests for Differences of Means (Minitab^®^ Statistical Software, version 19.2). The homoscedasticity of the dataset and the normality of residuals was visually checked prior to the analyses.

#### 2.3.4 Population structure

Population structure was investigated first by the analysis of the pairwise estimates of F_ST_ (Weir & Cockerham, 1984), and their significance were computed using FSTAT (Goudet, 2001). Second, a Bayesian clustering method as implemented in Structure 2.3.4 (Pritchard, Stephens, & Donnelly, 2000) was used to determine the existence of differentiated genetic groups within *L. digitata* populations categorizing them into K subpopulations. A range of clusters (K) from one to six was tested with 100 iterations, a burn‐in period of 100,000, and a Markov chain Monte Carlo of 500,000 (Gilbert et al., 2012). The most likely value of K was determined using Evanno ΔK (Evanno, Regnaut, & Goudet, 2005) obtained using Structure Harvester (Earl & vonHoldt, 2012). Replicates of Structure runs were combined using CLUMPP software (Jakobsson & Rosenberg, 2007). Bar plots were created with Distruct (Rosenberg, 2004).

## 3 RESULTS

### 3.1 Heat stress experiment

The significant main effects of independent factors are only reported in the absence of significant interactive effects. Therefore, in the presence of significant interactive effects, the simultaneous effects of two or more independent variables on a given dependent variable are given more emphasis than significant main effects.

#### 3.1.1 Growth

**Figure 3:**
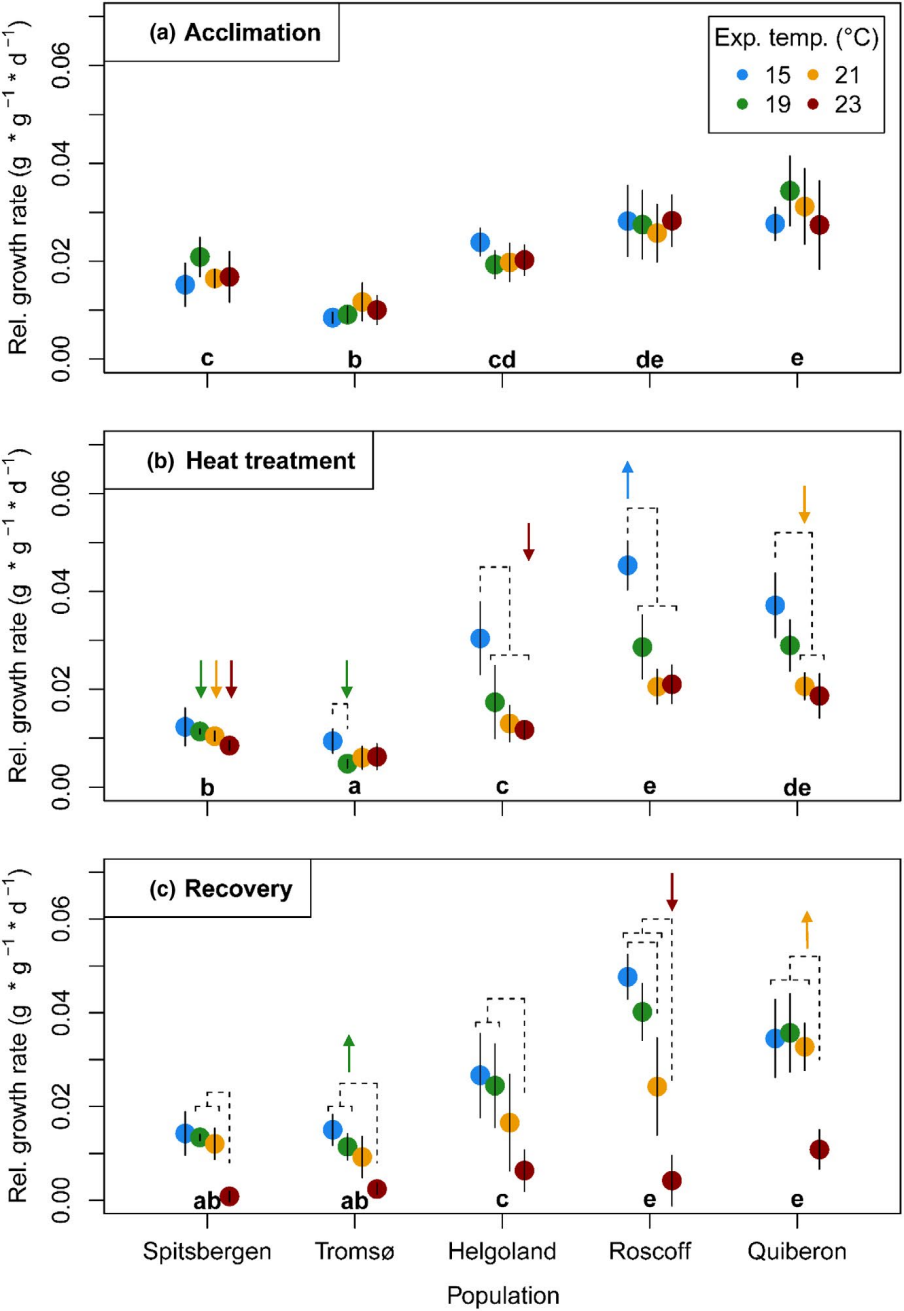
Relative growth rates of *Laminaria digitata* disks over the experimental phases of (a) acclimation at 15°C, (b) heat treatment, and (c) recovery at 15°C. Mean values ± *SD* (*n* = 5, for Spitsbergen *n* = 4). Lowercase letters indicate significant differences between all mean population responses over time (Bonferroni tests, *p* < .05). Dashed lines indicate significant differences between temperature treatments within populations (Bonferroni tests, *p* < .05). Arrows indicate significant differences between temperature treatments over time (Bonferroni tests, *p* < .05). Significance levels are given in the text

The significant population × temperature × time interaction for relative growth rates (Figure 3; Table 1) indicates that growth in the temperature treatments differed significantly between populations over exposure time. However, there were differences in general growth activity between populations already during acclimation at 15°C (Figure 3a), which persisted during the heat and recovery phases (Figure 3b,c), indicating a different physiological status among populations. This is represented by the significant main effect of population on growth rates (Figure 3; Table 1). Mean growth over all temperatures and time points was significantly lower in material from the northern populations Spitsbergen and Tromsø (by 34%–70%) than in material from the southern populations Helgoland, Roscoff, and Quiberon ((ROS = QUI) > HLG > SPT > TRO, Bonferroni‐corrected pairwise comparisons, *p < *.001).

**Table 1:** Results of generalized least squares models to examine variability of relative growth rates (RGR) and maximum quantum yield (F_v_/F_m_) of *Laminaria digitata* disks in the heat stress experiment

Parameter	numDF	denDF	RGR		F_v_/F_m_	
			*F*‐value	*p*‐value	*F*‐value	*p*‐value
Population	4	228	283.25	**<.0001**	36.77	**<.0001**
Temperature	3	228	60.38	**<.0001**	29.06	**<.0001**
Time	2	228	54.56	**<.0001**	104.37	**<.0001**
Population × temperature	12	228	12.13	**<.0001**	5.56	**<.0001**
Population × time	8	228	7.70	**<.0001**	8.09	**<.0001**
Temperature × time	6	228	31.83	**<.0001**	32.91	**<.0001**
Population × temperature × time	24	228	3.20	**<.0001**	5.58	**<.0001**

Note

Fresh weight relative growth rates and maximum quantum yield F_v_/F_m_ over acclimation, heat treatment, and recovery periods were tested against interactive effects of population, heat stress temperature treatment, and time. Tested values are means of 2 per replicate (*n* = 5, *n* = 4 for Spitsbergen). numDF, numerator degrees of freedom; denDF, denominator degrees of freedom. Statistically significant values are indicated in bold text.

During the heat stress treatment (Figure 3b), interactive effects of temperatures and populations became evident. While temperature effect sizes were small in the northern populations, possibly because of the generally low growth activity, growth rates of Helgoland, Roscoff, and Quiberon material at 21°C and 23°C were 50%–60% lower than at 15°C. In both Helgoland and Roscoff samples, 19°C–23°C significantly reduced growth compared to the 15°C control (Bonferroni test, *p < *.01), whereas samples from Quiberon grew significantly slower only at 21°C and 23°C compared to 15°C (Bonferroni tests, *p < *.001). Quiberon was the only population where growth did not decrease significantly at 19°C neither over time nor compared to the 15°C control.

Over the recovery period at 15°C (Figure 3c), specimens from all populations showed significantly decreased growth after exposure to 23°C compared to lower temperature treatments (Bonferroni tests, *p < *.05). Spitsbergen and Tromsø essentially ceased growth (RGR < 0.001 and 0.002 g g^−1^ day^−1^, respectively), while Helgoland, Roscoff, and Quiberon maintained slow growth (0.006, 0.004, and 0.01 g g^−1^ day^−1^, respectively). However, during recovery after exposure to 23°C, there were no significant differences between growth rates of the different populations (Bonferroni tests, *p > *.05). Quiberon material recovered best, in that there were no significant differences between the 15 and 21°C treatments while disks in these treatments simultaneously grew significantly faster than those from the former 23°C treatment (Bonferroni tests, *p < *.01).

**Figure A1:**
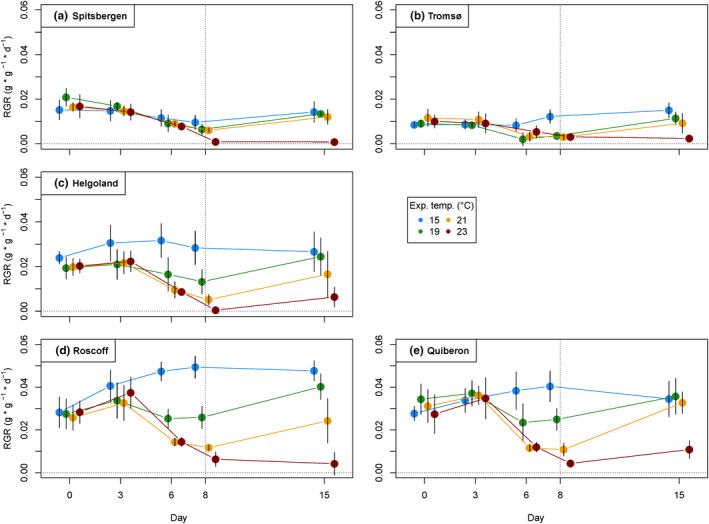
Relative growth rates (RGR) of *Laminaria digitata* disks from (a) Spitsbergen, (b) Tromsø, (c) Helgoland, (d) Roscoff, and (e) Quiberon over the heat stress experiment. Points represent growth rates between subsequent measuring days. Mean values ± SD (*n* = 5, for Spitsbergen *n* = 4). Points at day 0 represent growth over acclimation at 15°C, the end of the heat treatment at day 8 is marked with a vertical dotted line, and zero growth is marked with a horizontal dotted line. For statistical analysis, see Table A1.

In the more detailed time course of growth rates (Figure A1), it became evident that all populations showed a trend of recovery from 21°C as growth rates increased between day 8 and day 15 (Figure A1), which was significant only for Quiberon (RM ANOVA; Table A1; Bonferroni test, *p < *.001) and Spitsbergen (RM ANOVA; Table A1; Bonferroni test, *p < *.01). Additionally, only Helgoland and Quiberon material slightly, but not significantly, recovered growth rates from the 23°C treatment (RM ANOVAs; Table A1; Bonferroni tests, *p > *.05). At the end of the experiment, one Spitsbergen disk had bleached in the 23°C treatment, while all other disks survived.

#### 3.1.2 Photoacclimative responses

**Figure 4:**
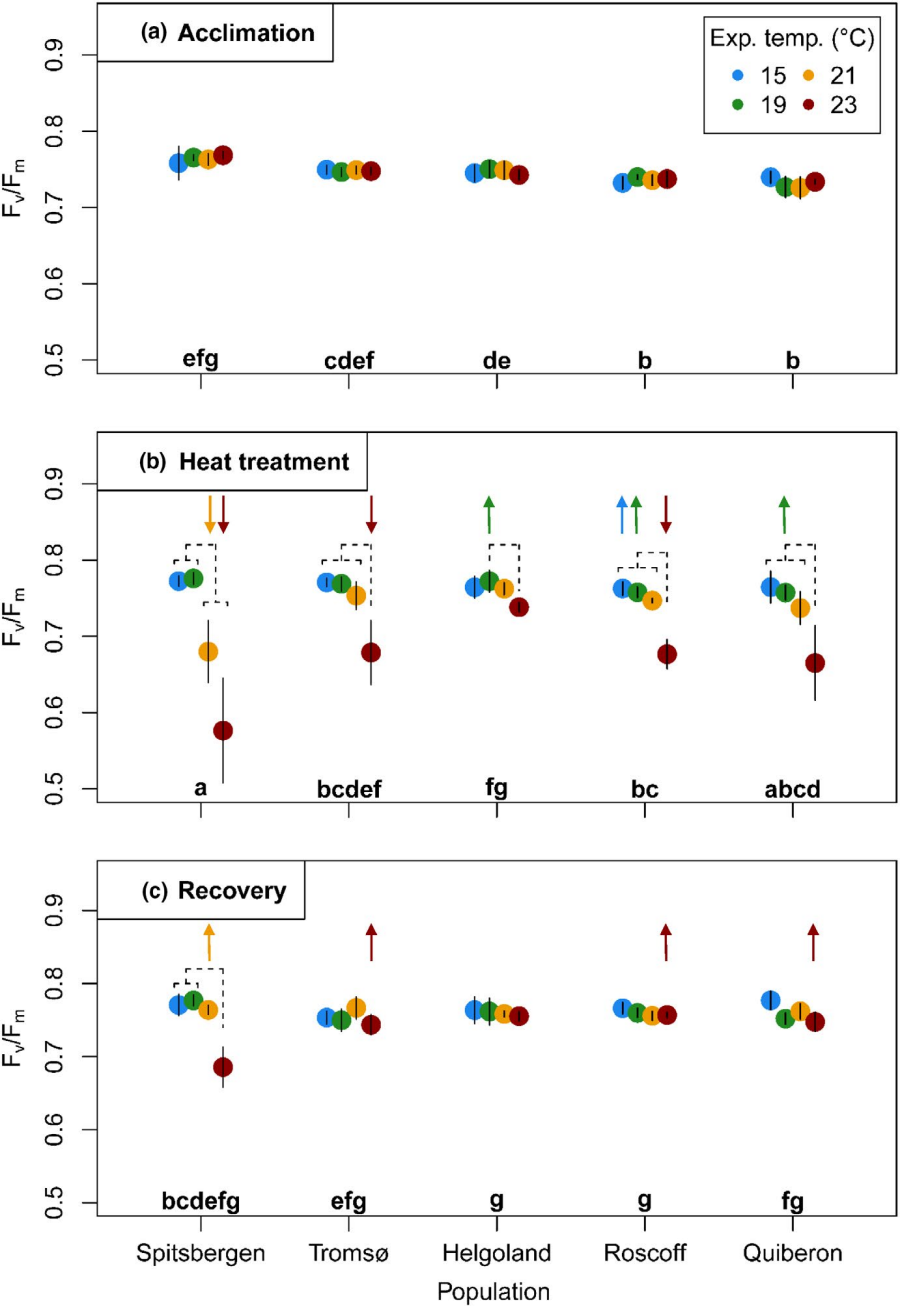
Maximum quantum yield (F_v_/F_m_) of *Laminaria digitata* disks after the experimental phases of (a) acclimation at 15°C, (b) heat treatment, and (c) recovery at 15°C. Mean values ± *SD* (*n* = 5, for Spitsbergen *n* = 4). Lowercase letters indicate significant differences between all mean population responses over time (Bonferroni tests, *p* < .05). Dashed lines indicate significant differences between temperature treatments within populations (Bonferroni tests, *p* < .05). Arrows indicate significant differences between temperature treatments over time (Bonferroni tests, *p* < .05). Significance levels are given in the text

Maximum quantum yield of photosystem II (F_v_/F_m_) in the temperature treatments differed between populations over time, which is represented by the significant population × temperature × time interaction (Figure 4, Table 1). After acclimation, all samples showed no signs of stress with F_v_/F_m_ ranging between 0.7 and 0.8 (Figure 4a).

At the end of the heat treatment (Figure 4b), temperature effects on quantum yield contrasted between the two populations of Spitsbergen and Helgoland. Spitsbergen material was most susceptible to the heat treatments: At 21°C and 23°C, quantum yield was significantly lower (by 12% and 25%, respectively) than at 15°C and 19°C (Bonferroni tests, *p < *.001). Tromsø, Roscoff, and Quiberon samples responded with a significant decrease in quantum yield by 11%–13% only at 23°C (Bonferroni tests, *p < *.05). In contrast, Helgoland samples were most stress resistant and showed a general stability of quantum yield in all conditions over time. Only at 23°C, at the end of the heat treatment, there was a slight decrease in quantum yield (significantly different only to the 19°C treatment; Bonferroni test, *p < *.001), but F_v_/F_m_ was still significantly higher (9%–28%) than in all other populations at 23°C (Bonferroni tests, *p < *.01).

**Figure A2:**
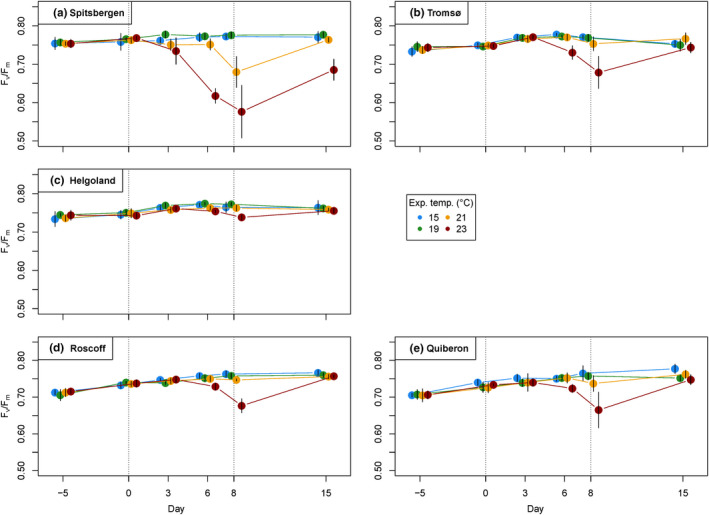
Maximum quantum yield (F_v_/F_m_) of *Laminaria digitata* disks from (a) Spitsbergen, (b) Tromsø, (c) Helgoland, (d) Roscoff, and (e) Quiberon over the heat stress experiment. Mean values ± SD (*n* = 5, for Spitsbergen *n* = 4). End of the acclimation at 15°C and end of the heat treatment are marked with dotted lines. For statistical analysis, see Table A1.

At higher temporal resolution (Figure A2), a general difference between southern and northern populations became more pronounced. While the significant decrease in quantum yield at 23°C took place between day 6 and day 8 for Helgoland, Roscoff, and Quiberon (RM ANOVA; Table A1; Bonferroni tests, *p < *.05), this decrease already started between day 3 and 6 in Spitsbergen and Tromsø material (Bonferroni tests, *p < *.001). Only specimens from Spitsbergen, as the most susceptible population, significantly decreased quantum yield also at 21°C, between day 6 and day 8 (Bonferroni test, *p < *.01).

The stronger heat susceptibility of Spitsbergen material became evident also following the recovery period (Figure 4c). While all other populations recovered from 23°C, in that there were no significant differences to the 15°C control, Spitsbergen only recovered successfully from 21°C (Bonferroni tests, *p > *.05). However, F_v_/F_m_ did not recover in Spitsbergen material following the 23°C treatment (compared to 15–19°C; Bonferroni tests, *p < *.01), indicating chronic photoinhibition and likely damage to photosystem II.

**Figure A3:**
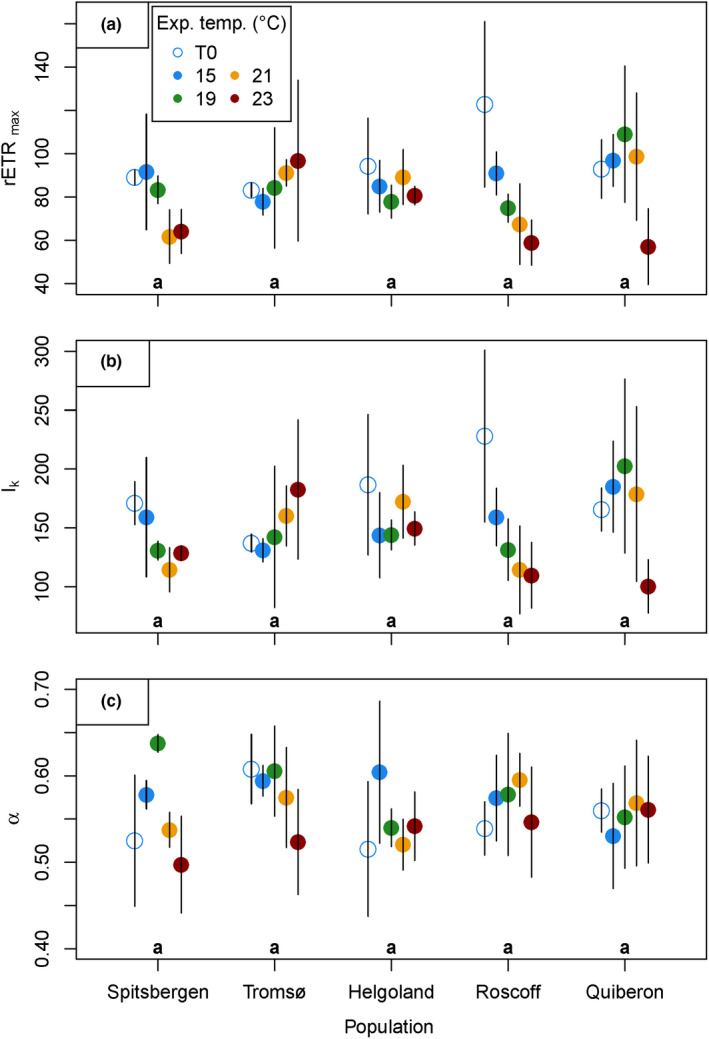
Photoacclimation parameters of *Laminaria digitata* disks obtained via rapid light curves after acclimation at 15°C (day 0, empty circles) and after the heat treatment (day 8, colored points). (a) Maximum relative electron transport rate rETR_max_ (relative unit), (b) saturation irradiance I_k_ (µmol photons m^−2^ s^−1^), (c) photosynthetic efficiency α (rETR/µmol photons m^−2^ s^−1^). Mean values ± SD (*n* = 3, for Spitsbergen *n* = 2). Analyses of variance returned no significant differences between populations (indicated by lowercase letters) and temperatures (Table A2).

**Figure A4:**
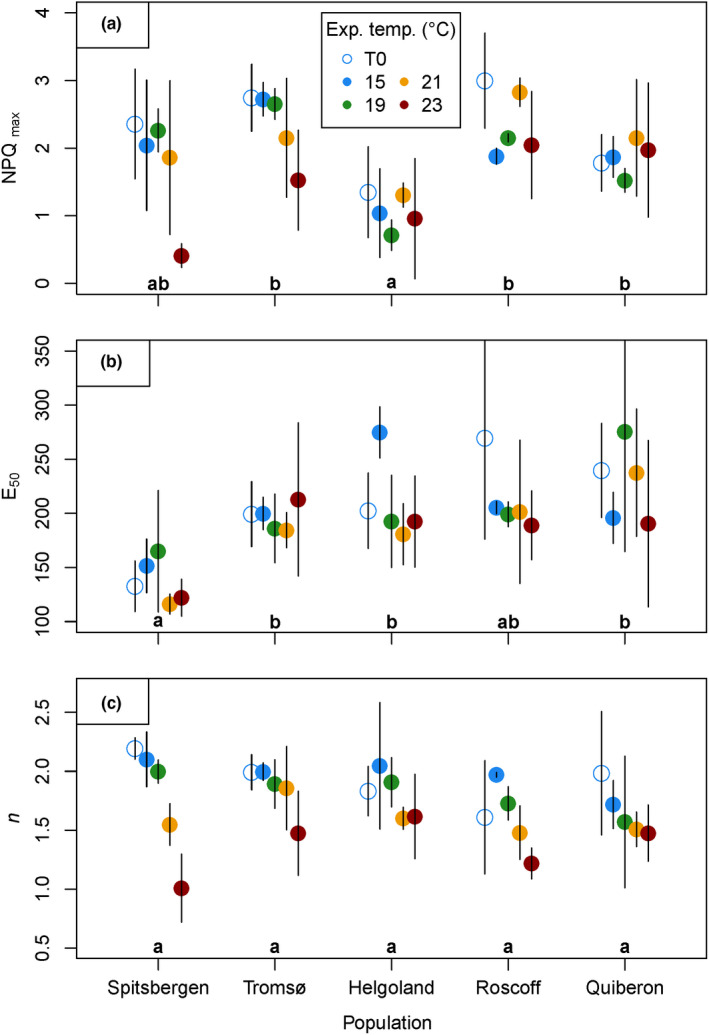
Nonphotochemical quenching parameters of *Laminaria digitata* disks obtained via rapid light curves after acclimation at 15°C (day 0, empty circles) and after the heat treatment (day 8, colored points). (a) Maximum nonphotochemical quenching NPQ_max_ (relative unit), (b) saturation irradiance E_50_ (µmol photons m^−2^ s^−1^), (c) sigmoidicity coefficient *n* (unitless). Mean values ± *SD* (*n* = 3, for Spitsbergen *n* = 2). Significant differences between mean population responses are indicated by lowercase letters (Table A3; Tukey tests, *p* < .05).

Contrary to quantum yield, the photoacclimation parameters obtained from rapid light curves at the end of the heat treatment, maximum relative electron transport rate rETR_max_ (Figure A3a), saturation irradiance I_k_ (Figure A3b), and photosynthetic efficiency α (Figure A3c) did not show significant effects or interactions of temperature and population (Table A2). In contrast, nonphotochemical quenching (NPQ) parameters showed no significant interaction effects, but significant effects of population on maximum nonphotochemical quenching NPQ_max_ and saturation irradiance E_50_, and of temperature on the sigmoidicity coefficient *n* (Figure A4; Table A3). Mean NPQ_max_ (Figure A4a) was 47%–56% lower in Helgoland material than in Tromsø, Roscoff, and Quiberon over all temperatures ((QUI = ROS =TRO = SPT) > (SPT = HLG); Tukey tests, *p < *.05), indicating intrinsically low nonphotochemical quenching in the Helgoland population. Mean E_50_ (Figure A4b) of Spitsbergen material was significantly lower than in Tromsø, Helgoland, and Quiberon by 29%–38% over all temperatures ((QUI = ROS =HLG = TRO) > (ROS = SPT); Tukey tests, *p < *.05), indicating an onset of NPQ already at low irradiances for Spitsbergen. The significant effect of temperature on *n* (Figure A4c) was visible as a mean downward trend of *n* by 29% between 15 and 23°C over all populations ((15°C = 19°C) > (19°C = 21°C) > (21°C = 23°C); Tukey tests, *p < *.001), indicating a greater response of NPQ under lower irradiances at high temperatures.

#### 3.1.3 Biochemistry

**Figure 5:**
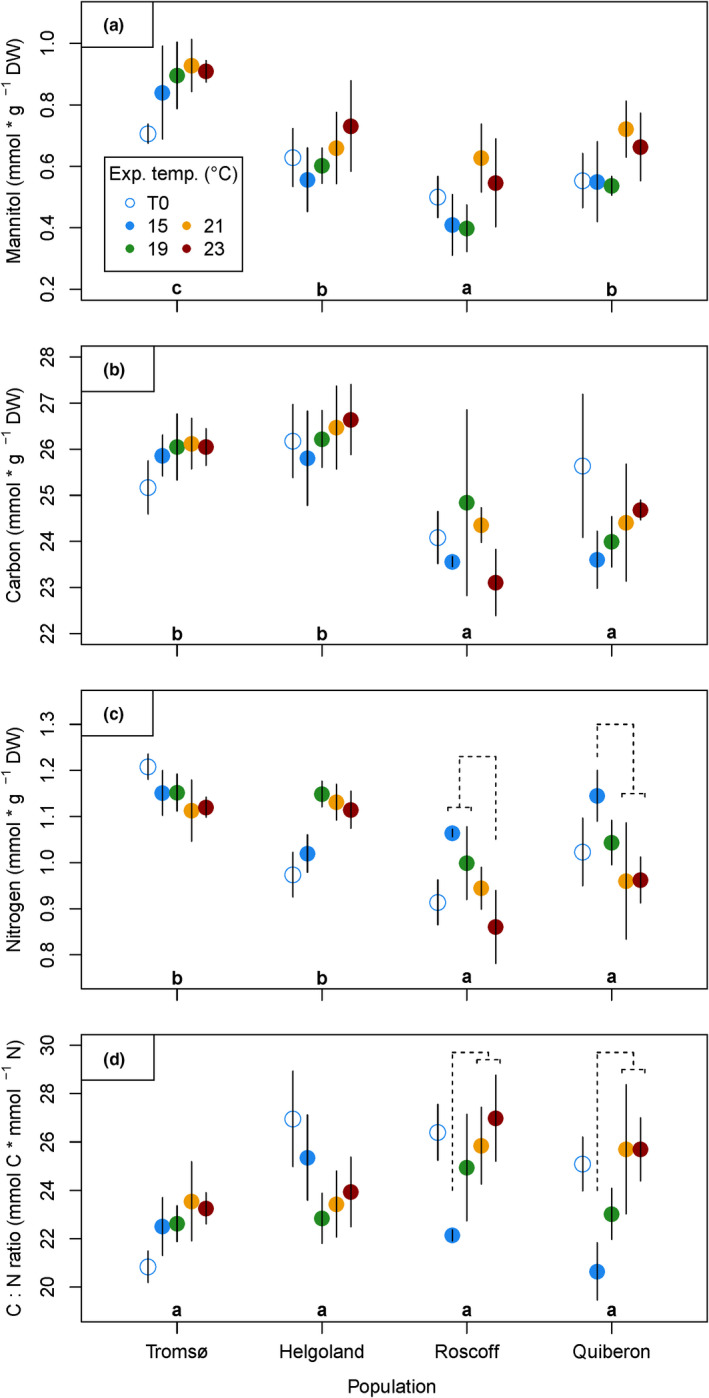
Biochemical characteristics of *Laminaria digitata* disks after acclimation at 15°C (day 0, empty circles) and after the heat treatment (day 8, colored points). (a) Mannitol contents, (b) carbon contents, (c) nitrogen contents, (d) molar C:N ratio. Mean values ± *SD* (*n* = 5, *n* = 4 for Quiberon in carbon, nitrogen, and C:N ratio), except for (a) means of mean values due to extraction in triplicates. Significant differences between mean population responses are indicated by lowercase letters (Tukey tests, *p* < .05). Significant differences between temperature treatments within populations are indicated by dashed lines (Tukey tests, *p* < .05). Significance levels are given in the text

Tissue mannitol and carbon contents were not significantly affected by interactive effects of population and temperature (Figure 5; Table 2), indicating that all populations responded uniformly to the temperature treatments in carbon storage. The significant effect of population on mannitol contents (Table 2) was due to the lowest contents in Roscoff and 80% higher contents in Tromsø material (TRO > (HLG = QUI) > ROS; Tukey tests, *p < *.05). The significant effect of temperature on mannitol (Table 2) shows that 21°C and 23°C induced significantly higher mannitol contents compared to the 15°C and 19°C treatments over all populations ((23°C = 21°C) > (19°C = 15°C); Tukey tests, *p < *.05). Carbon contents were not affected by temperature, but differed significantly only between populations (Figure 5b; Table 2). As with mannitol, Tromsø material maintained a higher carbon content, in that the means were significantly (7%–9%) higher in Tromsø and Helgoland material than in Roscoff and Quiberon material ((TRO = HLG) > (ROS = QUI); Tukey tests, *p < *.001).

**Table 2:** Results of generalized least squares models to examine variability of biochemical characteristics of *Laminaria digitata* disks in the heat stress experiment

Parameter	numDF	denDF	Mannitol		Carbon		Nitrogen		C:N ratio	
			*F*‐value	*p*‐value	*F*‐value	*p*‐value	*F*‐value	*p*‐value	*F*‐value	*p*‐value
Initial values	1	63 (59)	96.04	**<.0001**	65.82	**<.0001**	49.08	**<.0001**	8.56	**.0049**
Population	3	63 (59)	19.54	**<.0001**	42.76	**<.0001**	17.48	**<.0001**	2.93	**.0410**
Temperature	3	63 (59)	9.67	**<.0001**	2.46	.0718	7.78	**.0002**	8.63	**.0001**
Population × temperature	9	63 (59)	0.92	.5133	1.90	.0688	6.18	**<.0001**	4.82	**.0001**

Note

Molar mannitol content, carbon content, nitrogen content, and C:N ratio were tested against initial values as covariate and interactive effects of population and heat stress temperature treatment. *n* = 5, *n* = 4 for Quiberon in carbon, nitrogen, and C:N ratio. numDF, numerator degrees of freedom; denDF, denominator degrees of freedom. denDF = 59 for carbon, nitrogen, and C:N ratio. Statistically significant values are indicated in bold text.

Nitrogen contents were significantly affected by interactive effects of population and temperature (Figure 5c; Table 2). Only Roscoff and Quiberon samples showed a significant decrease in nitrogen contents at high temperatures (at 23°C for Roscoff, Tukey tests, *p < *.05; at 21°C and 23°C for Quiberon, Tukey tests, *p < *.001). Compared to the 15°C control, 23°C led to a reduction in nitrogen content by 20% in Roscoff and 15% in Quiberon samples. In a pattern reverse to that of nitrogen, molar C:N ratios were significantly affected by interactive effects of population and temperature (Figure 5d; Table 2). C:N ratios in the 21°C and 23°C treatments were therefore significantly higher than in the 15°C control for Roscoff and Quiberon samples (Tukey tests, *p < *.05).

The model covariate for initial values had a significant effect on all biochemical parameters taken at the end of the experiment (Table 2), in which higher initial values were correlated with higher values at the end of the heat treatment. Significant negative correlations of growth rates with mannitol (Kendall's tau = −0.5570; *p < *.0001; Table A4), carbon (Kendall's tau = −0.4218; *p < *.0001), and nitrogen contents (Kendall's tau = −0.2547, *p = *.0011) indicated growth at the expense of storage.

#### 3.1.4 Pigments

**Figure 6:**
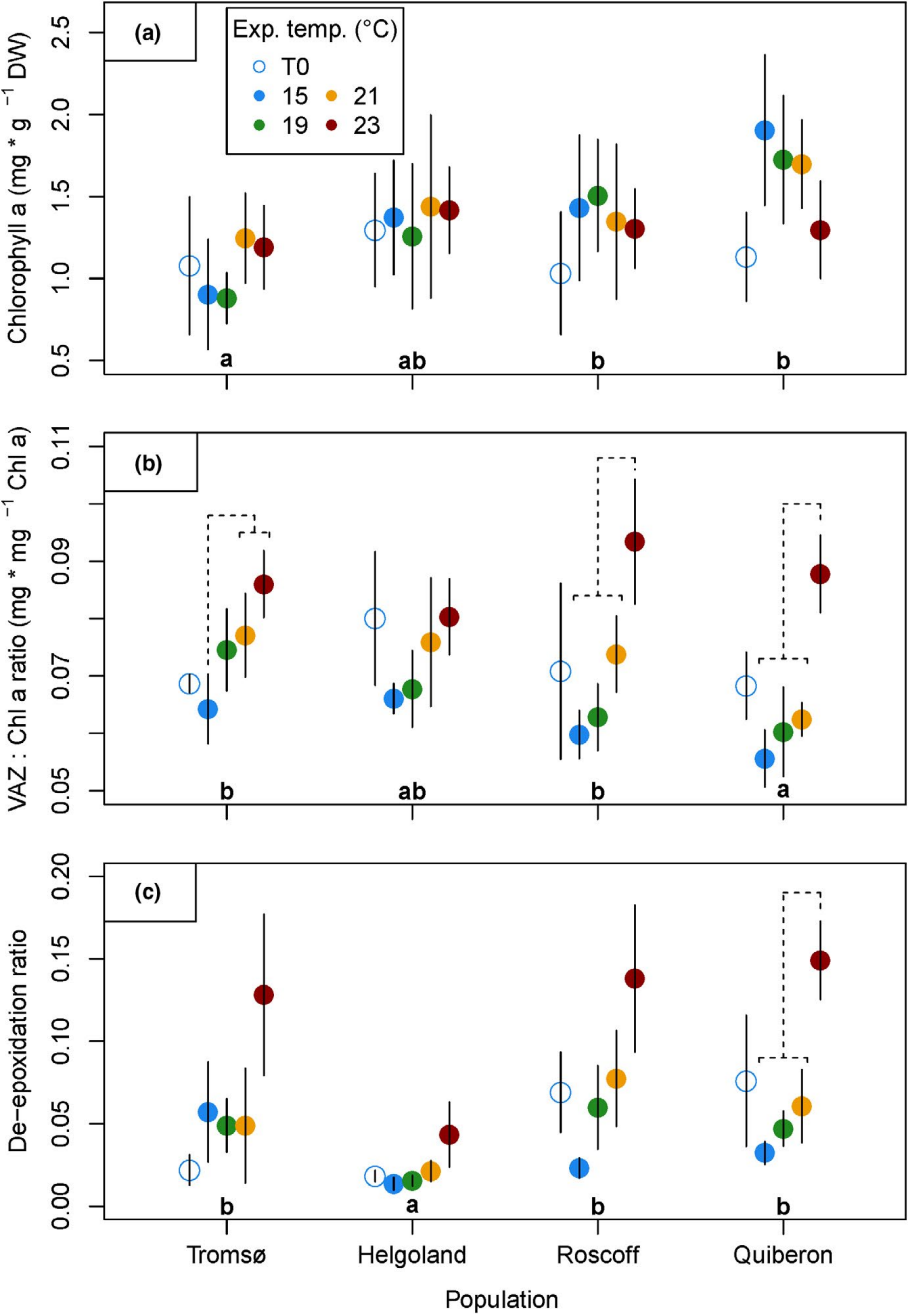
Pigment characteristics of *Laminaria digitata* disks after acclimation (day 0, empty circles) and after the heat treatment (day 8, colored points). (a) Chlorophyll *a* contents, (b) mass ratio of xanthophyll pigments per Chlorophyll *a* (VAZ : Chl *a* ratio), (c) de‐epoxidation ratio of xanthophyll pigments. Mean values ± *SD* (*n* = 5, *n* = 4 for Tromsø 23°C in VAZ : Chl *a* ratio and de‐epoxidation ratio). Significant differences between mean population responses are indicated by lowercase letters (Tukey tests, *p* < .05). Significant differences between temperature treatments within populations are indicated by dashed lines (Tukey tests, *p* < .05). Significance levels are given in the text

Chlorophyll *a* content was not significantly affected by interactive effects of population and temperature, but differed significantly between populations (Figure 6a; Table 3). Mean chlorophyll *a* contents were significantly (24%–36%) lower in Tromsø samples than in Roscoff and Quiberon material ((QUI = ROS = HLG) > (HLG = TRO); Tukey tests, *p < *.05), while chlorophyll *a* content in Helgoland material did not differ significantly from the other populations.

**Table 3:** Results of generalized least squares models to examine variability of pigment characteristics of *Laminaria digitata* disks in the heat stress experiment

Parameter	numDF	denDF	Chl *a*		VAZ : Chl *a* ratio		De‐epoxidation ratio	
			*F*‐value	*p*‐value	*F*‐value	*p*‐value	*F*‐value	*p*‐value
Initial values	1	63 (62)	1.22	.2731	22.95	**<.0001**	95.39	**<.0001**
Population	3	63 (62)	9.08	**<.0001**	3.53	**.0198**	22.96	**<.0001**
Temperature	3	63 (62)	0.53	.6653	51.39	**<.0001**	42.51	**<.0001**
Population × temperature	9	63 (62)	1.30	.2534	2.25	**.0298**	6.96	**<.0001**

Note

Chlorophyll *a* content, xanthophyll pigment (VAZ) : Chl *a* ratio, and de‐epoxidation ratio were tested against initial values as covariate and interactive effects of population and heat stress temperature treatment. *n* = 5, *n* = 4 for Tromsø 23°C in VAZ : Chl *a* ratio and de‐epoxidation ratio. numDF, numerator degrees of freedom; denDF, denominator degrees of freedom. denDF = 62 for VAZ : Chl *a* ratio and de‐epoxidation ratio. Statistically significant values are indicated in bold text.

The mass ratio of xanthophyll pigments per chlorophyll *a* (VAZ : Chl *a* ratio) was affected significantly by initial values, and interactive effects of population and temperature (Figure 6b; Table 3). Temperature had a significant, overall increasing effect on VAZ : Chl *a* ratios (23°C > 21°C > (19°C = 15°C), Tukey tests, *p < *.05), indicating accumulation of xanthophyll pigments as a photoprotective stress response toward temperature. Tromsø material significantly increased VAZ : Chl *a* ratios in the 21°C and 23°C treatments compared to the 15°C control (Tukey tests, *p < *.05) by 20% and 34%, respectively. A significant increase in VAZ : Chl *a* ratios became evident in the 23°C treatment compared to all other temperatures within the Roscoff (Tukey tests, *p < *.05) and Quiberon (Tukey tests, *p < *.01) populations. Compared to the 15°C control, 23°C led to an increase in VAZ : Chl *a* by more than 50% for both populations from Brittany, thereby presenting the strongest response in xanthophyll accumulation. In contrast, no significant differences between temperature treatments arose within the Helgoland population, further demonstrating a lack of heat stress response.

De‐epoxidation ratios of xanthophyll cycle pigments were affected significantly by initial values, and interactive effects of population and temperature (Figure 6c, Table 3). The significant differences between populations in mean de‐epoxidation ratios over all temperatures (Table 3) show that de‐epoxidation ratios were significantly lower in Helgoland samples than in all other populations ((QUI = ROS = TRO) > HLG; Tukey tests, *p < *.01). This result supports low values for nonphotochemical quenching in Helgoland material (NPQ_max_; Figure A4a). Overall, higher temperatures significantly increased de‐epoxidation ratios (23°C > (21°C = 19°C) > (19°C = 15°C), Tukey tests, *p < *.05). The highest temperature of 23°C led to a mean increase in the de‐epoxidation ratio by a factor of 2 in Tromsø, a factor of 3 in Helgoland, a factor of 6 in Roscoff, and a factor of 4.5 in Quiberon material compared to the respective 15°C controls. However, the only significant within‐population temperature response to 23°C emerged in the Quiberon samples (Tukey tests, *p < *.05), showing the most pronounced heat response in the southernmost population.

Chlorophyll *a* content was positively correlated with growth (Kendall's tau = 0.2013; *p = *.0082; Table A4), while growth rates and VAZ : Chl *a* ratios were strongly negatively correlated (Kendall's tau = −0.2911; *p = *.0001), indicating negative effects of the heat treatments and resulting stress responses on growth. F_v_/F_m_ after the heat treatment was strongly negatively correlated with VAZ : Chl *a* ratios (Kendalls tau = −0.2828; *p = *.0002) and to de‐epoxidation ratios (Kendall's tau = −0.3954; *p < *.0001), supporting the interpretation of xanthophyll‐derived parameters as photoprotective stress proxies. Additionally, de‐epoxidation ratios positively correlated with maximum nonphotochemical quenching NPQ_max_ (Kendall's tau = 0.2155, *p = *.0328), further emphasizing the relation of xanthophyll pigments and photoprotection.

### 3.2 Population genetics

#### 3.2.1 Microsatellite amplification

Null alleles were present in every population for at least two markers (Table A5). However, differences between F_ST_ values in the pairwise comparison were never greater than 10^–3^ (data not shown). Therefore, we concluded that the frequency of null alleles was negligible and our dataset was analyzed without taking into account correction for null alleles. No significant linkage disequilibrium was observed in any of the populations (Table A6). We thus considered all of the markers as independent. The number of alleles per locus ranged from 2 to 22 (Lo454‐27 and Ld371, respectively).

#### 3.2.2 Genetic diversity

Values of genetic diversity averaged over the 12 loci are provided in Table 4 for each population (for details of genetic diversity estimates locus by locus see Table A7). Most quantities varied by a factor of 1.5 among populations; the lowest genetic diversity was always observed in Helgoland and the highest in Roscoff. Variation was the highest for the mean number of private alleles (P_a_) which ranged from 0.083 to 0.583. The differences between populations were not significant when each parameter was tested independently (one‐way ANOVA, data not shown). However, a Fisher test of pairwise differences between means revealed that AR and P_a_ were significantly lower in Helgoland compared to Roscoff (data not shown). In addition, three of the twelve loci were monomorphic in Helgoland, compared to the other populations, in which a maximum of one monomorphic locus was observed (Table A7).

**Table 4:** Genetic characteristics of the *Laminaria digitata* populations used in the heat stress experiment

Population	Year	*n*	N_a_	AR	P_a_	H_e_	H_o_	F_IS_
Spitsbergen	2019	26	3.667 ± 0.620	3.494 ± 0.427	0.250 ± 0.131	0.436 ± 0.069	0.362 ± 0.058	0.127 ± 0.054
Tromsø	2018	30	3.583 ± 0.596	3.447 ± 0.422	0.250 ± 0.131	0.363 ± 0.074	0.350 ± 0.073	0.051 ± 0.055
Bodø	2018	32	4.833 ± 1.065	4.464 ± 0.699	0.500 ± 0.195	0.444 ± 0.088	0.376 ± 0.077	0.117 ± 0.033 *
Helgoland	2016	35	2.833 ± 0.638	2.594 ± 0.422	0.083 ± 0.083	0.306 ± 0.076	0.296 ± 0.078	0.039 ± 0.032
Roscoff	2018	28	5.167 ± 1.120	4.875 ± 0.786	0.583 ± 0.229	0.480 ± 0.082	0.429 ± 0.083	0.171 ± 0.044 *
Quiberon	2018	28	4.583 ± 0.773	4.186 ± 0.511	0.333 ± 0.142	0.432 ± 0.061	0.408 ± 0.067	0.106 ± 0.035

Note

Year: year of the samples used for genetic analysis (except for Helgoland, the genotyped individuals are the same than those analyzed for the heat stress experiment); *n*, number of individuals for which at least 11 markers amplified; N_a_, mean number of observed alleles; AR, allelic richness standardized for equal sample size (21 individuals); P_a_, mean number of private alleles per locus; H_e_, expected heterozygosity; H_o_, observed heterozygosity; F_IS_, fixation index (inbreeding coefficient) of individuals with respect to local subpopulation. All parameters are expressed as means over all markers ± standard error. *, significant departure from random mating after correction for multiple testing (*p* < .0069, FSTAT).

#### 3.2.3 Genetic structure

**Figure 7:**
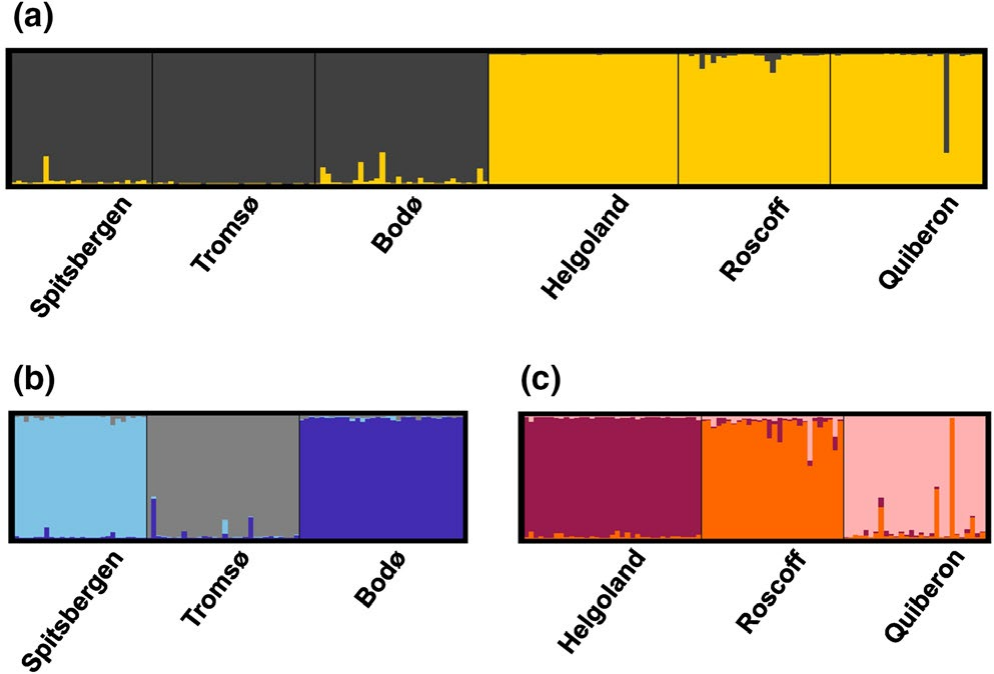
Structure bar plot of *Laminaria digitata* populations along the entire distribution range. (a) First hierarchical level of structure obtained for K = 2 genetic clusters. (b) Second hierarchical level of structure for northern populations and (c) second hierarchical level of structure for southern populations of *L. digitata* obtained for K = 3 genetic clusters. Individuals (vertical bars) were assigned probabilities of belonging to clusters (colors) based on differences in genetic variance

**Figure A5:**
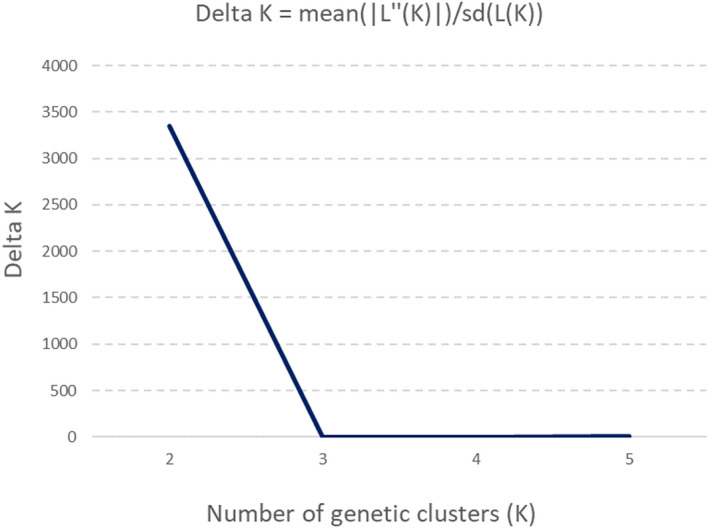
ΔK (Evanno et al., 2005) plotted against K, associated with K = 2 to K = 5 obtained with Structure Harvester during the analysis of genetic structure of *Laminaria digitata* populations

Genetic differentiation was significant for each pairwise population comparison (*p* = .003 for all pairs; FSTAT) with an average F_ST_ value of 0.3795 (Table A8), while the strongest differentiation occurred between Helgoland and Tromsø and the weakest between Helgoland and Roscoff. Structure analyses results show that the optimal number of genetic clusters was K = 2 according to the method of Evanno et al. (2005) (Figure A5). We detected a clear hierarchical distinction in genetic structure between two groups (Figure 7a) of northern populations (Spitsbergen, Tromsø, Bodø) and southern populations (Helgoland, Roscoff, Quiberon). A subsequent analysis run separately for northern and southern populations revealed distinct structuring between the three populations present in each subset (Figure 7b,c; K = 3). While gene flow between populations is generally very weak, the relatively highest connectivity occurred between the adjacent Roscoff and Quiberon populations. Additionally, a difference between northern and southern populations is visible at Lo454‐27 (Table A7), where one allele is fixed for all southern populations.

#### 3.2.4 Reproductive system

*L. digitata* from Tromsø and Helgoland did not show any significant departure from random mating (F_IS_). We identified F_IS_ > 0.1 for Spitsbergen, Bodø, Roscoff, and Quiberon, (Table 4 for multilocus estimates of F_IS_; Table A7 for single locus estimates of F_IS_). However, when *p*‐values were corrected for multiple testing (*p < *.0069, FSTAT), heterozygote deficiency was significant only for Bodø and Roscoff.

## 4 DISCUSSION

We identified a uniform growth limit across European *Laminaria digitata* populations following a short‐term application of 23°C, which conforms with previous studies (Bolton & Lüning, 1982; tom Dieck, 1992). Despite this, we observed slight deviations in magnitude and onset of stress responses among *L. digitata* populations at the cold and warm range margins. Arctic Spitsbergen material presented the strongest heat stress reaction. On the other hand, the two populations naturally experiencing summer temperatures near their upper long‐term survival limit, Helgoland and Quiberon, showed moderate advantages in stress responses and growth during the heat treatments. We therefore provide further evidence for the existence of thermal ecotypes of *L. digitata* (King et al., 2019) across the species’ entire Northeast Atlantic distribution. The strong genetic structuring of *L. digitata* within northern and southern clades might have facilitated phenotypic divergence, while neutral genetic diversity was not connected to clear patterns of genetic drift or maladaptation along *L. digitata*'s latitudinal distribution.

### 4.1 Similarities in growth and biochemical responses along the latitudinal gradient

Growth responses among our tested populations suggest that the upper temperature tolerance limit of *Laminaria digitata* is uniform along its European latitudinal distribution. Growth is an integrative parameter of all metabolic processes and can thus be interpreted as a proxy for organismal stress response. We observed that growth almost completely ceased in the 23°C treatment for all populations (Figure 3), while all populations showed signs of recovery from 21°C when transferred to 15°C (Figure A1). The populations of Tromsø and Spitzbergen showed significantly lower overall growth rates than the southern populations. The lower growth rates of the Arctic populations might be related to prevailing local environmental conditions during sampling (e.g., long day lengths, cold temperature) which may influence growth rates and circannual rhythmicity in kelps (Olischläger & Wiencke, 2013; Schaffelke & Lüning, 1994). Still, results of our study using meristematic disks of wild adult *L. digitata* material support previous studies using laboratory‐cultivated whole juvenile *L. digitata* sporophytes, which also showed uniform upper temperature limits on both sides of the Atlantic and Spitsbergen (Bolton & Lüning, 1982; Franke, 2019; tom Dieck, 1992).

The definition of thermal limits across populations strongly depends on the experimental design (e.g., cultivation conditions and sample age, among other independent variables) and on the response variables measured. Previous studies using photosynthesis (Helgoland: Lüning, 1984) and tissue damage (Nova Scotia: Simonson et al., 2015) as proxies defined the upper thermal tolerance of wild *L. digitata* sporophytes at 18°C to 20°C in experiments that lasted one week. Higher temperatures of 21°C (Simonson et al., 2015) and 23°C (Lüning, 1984) were lethal. However, common‐garden experiments demonstrated the capacity for cultivated and wild juvenile *L. digitata* sporophytes from these locations to survive temperatures >20°C for at least one week using growth and occurrence of tissue bleaching as proxies (Helgoland and Nova Scotia: Bolton & Lüning, 1982; tom Dieck, 1992; Nova Scotia: Wilson et al., 2015). Physiological responses may also differ depending on the treatment duration. Whereas maximum quantum yield (F_v_/F_m_) of Southern English *L. digitata* decreased over a period of 16 days at 18°C (Hargrave et al., 2017), F_v_/F_m_ was stable at 19°C over a shorter period of seven days in our experiment. Still, the reduced growth at 18°C in Hargrave et al. (2017) matches the decrease in growth at 19°C in our study. Thus, uniformity or differences in thermal limits among populations can only be reliably assessed under common‐garden conditions, for example, as performed here.

In addition to the strong similarities in the upper thermal limits of growth in our study, carbon contents (Figure 5b) and chlorophyll *a* contents (Figure 6a) did not differ between temperature treatments at all. In contrast, the overall trend of increasing mannitol contents at high temperatures (Figure 5a) has been described for *Saccharina latissima* (Davison & Davison, 1987) and might be linked to the seasonal increase in kelp mannitol storage in summer during the period of slow growth (Haug & Jensen, 1954; Schiener et al., 2015), which, in wild sporophytes, is followed by a peak of the long‐term storage compound laminarin in autumn (Haug & Jensen, 1954; Schiener et al., 2015).

The consistent responses of growth and biochemical contents across populations reported here indicate a strong acclimation potential of *L. digitata'*s metabolism to high temperature. Acclimation to wide temperature ranges would reduce selective pressure of temperature in the wild and might explain the small magnitude of local differentiation observed in this study.

### 4.2 Differences in growth and photosynthetic parameters among marginal populations

Despite the stability of the upper thermal growth limit, we observed subtle physiological differences in the common‐garden heat stress experiment, mainly in the marginal populations of Spitsbergen, Helgoland, and Quiberon. Maximum quantum yield of photosystem II was most sensitive to thermal stress at 21°C and 23°C in Spitsbergen material (Figure 4; Figure A2). This is concordant with the subarctic to Arctic regional climate and provides first evidence for a loss of function in a leading‐edge *L. digitata* population, but whether this represents an adaptive trait is yet unknown. Generally, very few cold‐temperate algae occurring in the Arctic show true adaptations to the Arctic climate compared to their Atlantic populations (Bischoff & Wiencke, 1993; Wiencke et al., 1994), possibly because the Arctic did not provide a sufficiently stable environment for adaptive evolutionary processes to occur (Wiencke et al., 1994).

At the southern range edge, a slight advantage of Quiberon material to grow at elevated temperatures became evident in the growth response at 19°C during the heat treatment, and in the full recovery from the 21°C treatment (Figure 3; Figure A1). In contrast, photoacclimative responses suggest that the marginal population on the island of Helgoland was most resistant to heat stress. Photosystem II of Helgoland material was minimally impaired by 23°C (Figure 4). Additionally, reactions of xanthophyll pigments (Figure 6b,c) were significantly weaker in Helgoland material than other populations. Increased xanthophyll contents may indicate a photoprotective acclimation reaction (Latowski, Kuczyńska, & Strzałka, 2011; Pfündel & Bilger, 1994; Uhrmacher et al., 1995), while the de‐epoxididation ratio of xanthophyll cycle pigments represents the current capacity to quench excessive energy from the photosystem (Pfündel & Bilger, 1994). Helgoland material did not show a significant increase in xanthophyll pigments and presented significantly lower de‐epoxidation ratios and therefore lower nonphotochemical quenching (NPQ_max_, Figure A4) than all other populations. Therefore, the two populations growing in the warmest of the tested locations, which may experience >4 week long periods of mean in situ temperatures of 18°C to 19°C in summer (Helgoland: Bartsch, Vogt, Pehlke, & Hanelt, 2013; Wiltshire et al., 2008; Quiberon: Oppliger et al., 2014; Valero, unpubl.), showed slight physiological advantages to short‐term heat exposure in growth and stress responses.

The southernmost populations of Quiberon and Roscoff were curiously the only populations with significantly reduced tissue nitrogen contents in the heat treatments (Figure 5c). A variety of factors including temperature affects nutrient uptake and consequently tissue nitrogen contents, which could be species‐specific (Roleda & Hurd, 2019). Therefore, published studies on the impacts of heat stress on nitrogen uptake and storage in kelps differ in their reports of decreased (Gerard, 1997), unaffected (Nepper‐Davidsen et al., 2019), or increased nitrogen contents (Wilson et al., 2015). Whether the underlying cause of reduced nitrogen during heat in our study is adaptive, maladaptive, or neutral toward heat resilience in the southern populations remains unclear until further investigation.

### 4.3 Population genetics in relation to physiological thermal responses

Population genetics suggest that the slight phenotypic divergence of *L. digitata* might have been facilitated through phylogeographic separation into two clades and low genetic connectivity between populations. The hierarchical division into a northern and a southern clade in the Northeast Atlantic (Figure 7a) is likely due to postglacial recolonization by two distinct genetic groups located in refugia proposed for the Armorican/Celtic Sea (Brittany and South West UK) and a potential northern refugium at the west coast of Ireland and/or Scotland (Neiva et al., 2020; see also King et al., 2020). Currently, the highest genetic diversity (H_e_ ≥ 0.6) published for *L. digitata* populations was observed in Scotland (King et al., 2019, 2020), Northwest Ireland (Neiva et al., 2020), and Northeast Ireland (Brennan et al., 2014), which all exceeded the genetic diversity of the populations investigated in this study. Due to a lack of data, it remains unclear whether a potential glacial refugium of *L. digitata* also corresponds to the well‐described Southwest Ireland refugium proposed for many marine species (Kettle, Morales‐Muñiz, Roselló‐Izquierdo, Heinrich, & Vøllestad, 2011; Provan & Bennett, 2008).

Populations at the “leading edge” (high latitude) are said to be associated with low genetic diversity due to recolonization processes following the Last Glacial Maximum (Hampe & Petit, 2005; for marine seaweeds of the North Atlantic see Assis, Serrão, Claro, Perrin, & Pearson, 2014; Neiva et al., 2016; Provan & Maggs, 2012). Therefore, effects of genetic drift (e.g., depleted genetic diversity, increased inbreeding) may be expected to reduce physiological function in these populations. Here, genetic diversity characteristics of *L. digitata* at its northern range limit (i.e., Spitsbergen) were not significantly lower compared to the other populations in this study and were similar to other Northern Norwegian populations (Neiva et al., 2020). A similar pattern was observed for another Arctic to cold‐temperate kelp species, *Saccharina latissima* (Guzinski, Mauger, Cock, & Valero, 2016). Therefore, rather than effects of genetic drift, a lack of selection pressure in the Arctic might have led to a potential reduction of heat tolerance at the northern distribution limit (i.e., relaxed selection; Lahti et al., 2009; Zhen & Ungerer, 2008).

Probably due to the continuous rocky substrata along the Brittany coast, connectivity may be maintained between Quiberon and neighboring populations, which may explain a certain level of gene flow between Roscoff and Quiberon via stepping stone habitats (Figure 7c). Low gene flow can reduce inbreeding depression and associated deleterious effects and may facilitate local adaptation at this southern range edge (Fitzpatrick & Reid, 2019; Sanford & Kelly, 2011). Genetic diversity characteristics for Brittany *L. digitata* populations in this study comply with previous reports (Oppliger et al., 2014; Robuchon et al., 2014). Compared to Roscoff, genetic diversity of *L. digitata* from the island of Helgoland was significantly lower. The population's reduced genetic diversity can be partly explained by genetic isolation due to habitat discontinuity as Helgoland is a rocky substrate surrounded by continuous sandy seafloor (Reichert, Buchholz, & Giménez, 2008). This may rather suggest maladaptation due to less effective selection (such as in *Fucus serratus*; Pearson et al., 2009). However, samples from Helgoland presented the weakest heat stress response in this study. Therefore, we can hypothesize either that historically greater diversity/connectivity was reduced via isolation and drift after resilience to local conditions was established, or that strong selective forces toward the upper thermal limit of *L. digitata* have counterbalanced the effect of genetic drift.

Significant departures from random mating were only observed for the populations of Bodø and Roscoff (F_IS_; Table 4) and match the magnitude of recent descriptions for *L. digitata* populations (King et al., 2020; Neiva et al., 2020). The higher F_IS_ values in Roscoff *L. digitata* in our study compared to the nearby population of Santec (Robuchon et al., 2014) might be explained by the distance of >1 km between sites, which may already cause substantial variation in F_IS_ (Billot, Engel, Rousvoal, Kloareg, & Valero, 2003). In contrast, the higher F_IS_ values of Quiberon *L. digitata* in our study compared to Oppliger et al. (2014) who sampled at the same location (Pointe de Conguel North) may be an artifact of differing microsatellite markers or might indicate a change in the reproductive system over time (Oppliger et al., 2014; Valero et al., 2011). In all cases, in the absence of data on reproductive ecology, the underlying causes remain speculative.

### 4.4 Outlook

The mechanistic temperature treatments applied in this study do not represent realistic temperature scenarios for all tested populations, especially not for the northern clade. However, during our sampling period in August 2018, acute heat spikes surpassed 20°C on twelve days on Helgoland, and on nine days in Quiberon in the shallow sublittoral (in situ data; Bartsch, unpubl.; Valero, unpubl.). Also in South England, *L. digitata* already encounters marine heatwaves reaching 20°C (Burdett, Wright, & Smale, 2019; Joint & Smale, 2017). According to predictions of ocean warming (Müller et al., 2009) and marine heatwaves (Oliver et al., 2018), *L. digitata* will possibly encounter prolonged summer periods of 21°C–23°C at its warm distribution limit until the end of the century.

As a low intertidal to shallow sublittoral species, *L. digitata* is not only threatened by increasing summer SST and marine heatwaves (Bartsch et al., 2013; Hargrave et al., 2017), but also by other stressors during emersion such as desiccation and warm air temperature (Hereward, King, & Smale, 2020; King, Wilcockson, et al., 2018), high irradiance, and UV radiation (Gruber, Roleda, Bartsch, Hanelt, & Wiencke, 2011; Roleda, Hanelt, & Wiencke, 2006). These multiple stressors are most likely responsible for the die‐off event of Helgoland *L. digitata* sporophytes after exposure to SST > 19°C over a prolonged period of 11 days (Bartsch et al., 2013). Additionally, rising temperatures may negatively affect sporophyte and gametophyte reproduction (sporogenesis: Bartsch et al., 2013; gametogenesis: Lüning, 1980; Martins, Tanttu, Pearson, Serrão, & Bartsch, 2017) which might contribute to range contractions of *L. digitata*. Therefore, despite the slight physiological advantages we identified in the southern populations, *L. digitata* is threatened by a substantial loss of genetic diversity at its current southern distribution limit (King et al., 2020; Neiva et al., 2020; Oppliger et al., 2014; Robuchon et al., 2014).

Models have predicted a northward shift of the entire distribution range of *L. digitata* until 2100 in the RCP 8.5 emission scenario, implying possible extinction of southern populations, including Roscoff and Quiberon (Assis et al., 2018; Raybaud et al., 2013). A potential loss of more heat‐tolerant populations at the trailing edge and a simultaneous expansion of northern, slightly less heat‐tolerant *L. digitata* phenotypes implies that global warming might drive a decrease in the overall adaptive capacity to warming in the kelp *Laminaria digitata*. Neutral genetic diversity was recently described as an indicator for heat resilience of kelp populations by indicating physiological versatility among individuals (Wernberg et al., 2018). Conversely, marine heatwaves can deplete the genetic diversity of kelp populations in strong selective bottleneck events (Gurgel, Camacho, Minne, Wernberg, & Coleman, 2020). Therefore, response variability among individuals shapes the adaptive capacity of populations to withstand bottleneck events and to allow directional selection (Chevin, Lande, & Mace, 2010; Kelly, 2019; King, McKeown, et al., 2018). This implies that genetically depleted populations (e.g., marginal populations) are at even higher risk of extinction. A recent study showed high phenotypic variation among five genotypes of Helgoland *L. digitata* (Liesner et al., 2020), but studies correlating inter‐individual response variation to genetic diversity across populations are necessary to investigate the implications of genetic diversity for population resilience during climate change.

## CONFLICT OF INTEREST

All authors declare that they are free of competing interests.

## AUTHOR CONTRIBUTION

**Daniel Liesner:** Conceptualization (supporting); Data curation (equal); Formal analysis (lead); Investigation (lead); Methodology (supporting); Project administration (supporting); Visualization (lead); Writing‐original draft (lead). **Louise Fouqueau:** Data curation (equal); Formal analysis (supporting); Investigation (supporting); Visualization (supporting); Writing‐original draft (supporting); Writing‐review & editing (equal). **Myriam Valero:** Data curation (equal); Formal analysis (supporting); Funding acquisition (equal); Resources (supporting); Writing‐review & editing (equal). **Michael Y. Roleda:** Conceptualization (supporting); Formal analysis (supporting); Writing‐review & editing (equal). **Gareth A. Pearson:** Conceptualization (supporting); Formal analysis (supporting); Writing‐review & editing (equal). **Kai Bischof:** Resources (supporting); Supervision (supporting); Writing‐review & editing (equal). **Klaus Valentin:** Funding acquisition (equal); Resources (supporting); Supervision (supporting); Writing‐review & editing (equal). **Inka Bartsch:** Conceptualization (lead); Formal analysis (supporting); Funding acquisition (equal); Investigation (supporting); Project administration (lead); Resources (lead); Supervision (lead); Writing‐review & editing (equal).

## Data Availability

Genotype and physiological data generated during this study are available at platform DRYAD. Genotype data are accessible at https://doi.org/10.5061/dryad.jsxksn06c. Physiological data are accessible at https://doi.org/10.5061/dryad.73n5tb2ts.

## Appendix 1

### 1.1

See Tables A1, A2, A3, A4, A5, A6, A7, A8

**Table A1:** Results of generalized least squares models to examine variability of growth rates and maximum quantum yield of *Laminaria digitata* disks in the detailed time course of the heat stress experiment (Figures A1, A2)

Population	Parameter	RGR				F_v_/F_m_			
		numDF	denDF	*F*‐value	*p*‐value	numDF	denDF	*F*‐value	*p*‐value
Spitsbergen	Temperature	3	60	17.43	**<.0001**	3	72	46.35	**<.0001**
	Time	4	60	72.81	**<.0001**	5	72	9.31	**<.0001**
	Temperature × time	12	60	5.24	**<.0001**	15	72	14.78	**<.0001**
Tromsø	Temperature	3	80	87.90	**<.0001**	3	96	8.87	**<.0001**
	Time	4	80	778517.93	**<.0001**	5	96	45.61	**<.0001**
	Temperature × time	12	80	29.20	**<.0001**	15	96	7.02	**<.0001**
Helgoland	Temperature	3	80	159.35	**<.0001**	3	96	209.26	**<.0001**
	Time	4	80	23.36	**<.0001**	5	96	9198.09	**<.0001**
	Temperature × time	12	80	27.92	**<.0001**	15	96	7.47	**<.0001**
Roscoff	Temperature	3	80	168.27	**<.0001**	3	96	15.32	**<.0001**
	Time	4	80	22.71	**<.0001**	5	96	71.51	**<.0001**
	Temperature × time	12	80	14.12	**<.0001**	15	96	12.45	**<.0001**
Quiberon	Temperature	3	80	271.81	**<.0001**	3	96	14.08	**<.0001**
	Time	4	80	813970892.83	**<.0001**	5	96	67.21	**<.0001**
	Temperature × time	12	80	75.62	**<.0001**	15	96	4.34	**<.0001**

Notes

Fresh weight relative growth rates and maximum quantum yield F_v_/F_m_ over all time points (T‐5 (only F_v_/F_m_), T0, T3, T6, T8, T15) were tested against interactive effects of heat treatment and time for each population separately. Generalized least squares models were performed as described in the methods section, but without the fixed effect for population. Tested values are means of 2 per replicate (*n* = 5, *n *= 4 for Spitsbergen). numDF, numerator degrees of freedom; denDF, denominator degrees of freedom. Statistically significant values are indicated in bold text.

**Table A2:** Results of generalized least squares models to examine variability of photoacclimation parameters of *Laminaria digitata* disks obtained via rapid light curves in the heat stress experiment (Figure A3)

Parameter	numDF	denDF	rETR_max_		I_k_		α	
			*F*‐value	*p*‐value	*F*‐value	*p*‐value	*F*‐value	*p*‐value
Initial values	1	35	0.49	.4877	0.03	.8571	0.54	.4665
Population	4	35	2.32	.0760	2.25	.0831	0.39	.8149
Temperature	3	35	2.87	.0503	0.59	.6284	1.83	.1601
Population × temperature	12	35	1.59	.1413	1.38	.2197	1.04	.4390

Notes

Maximum relative electron transport rate rETR_max_, saturation irradiance I_k,_ and photosynthetic efficiency α were tested against initial values as covariate and interactive effects of population and heat stress temperature treatment. *n* = 3, *n *= 2 for Spitsbergen. numDF, numerator degrees of freedom; denDF, denominator degrees of freedom. Statistically significant values are indicated in bold text.

**Table A3:** Results of generalized least squares models to examine variability of nonphotochemical quenching parameters of *Laminaria digitata* disks obtained via rapid light curves in the heat stress experiment (Figure A4)

Parameter	numDF	denDF	NPQ_max_		E_50_		*n*	
			*F*‐value	*p*‐value	*F*‐value	*p*‐value	*F*‐value	*p*‐value
Initial values	1	35	78.00	**<.0001**	8.60	**.0059**	0.47	.4980
Population	4	35	20.73	**<.0001**	4.73	**.0037**	2.07	.1063
Temperature	3	35	1.68	.1883	2.14	.1132	11.43	**<.0001**
Population × temperature	12	35	1.86	.0761	1.17	.3397	0.91	.5448

Notes

Maximum nonphotochemical quenching NPQ_max_, saturation irradiance E_50,_ and sigmoidicity coefficient *n* were tested against initial values as covariate and interactive effects of population and heat stress temperature treatment. *n* = 3, *n* = 2 for Spitsbergen. numDF, numerator degrees of freedom; denDF, denominator degrees of freedom. Statistically significant values are indicated in bold text.

**Table A4:** Correlation coefficients (Kendall’s rank correlation tau) and *p*‐values in parentheses between relative growth rates (RGR), maximum quantum yield (F_v_/F_m_), biochemical, and pigment characteristics of *Laminaria digitata* during / after the heat treatment.

	*F_v_*/*F_m_*	Mannitol	Carbon	Nitrogen	C:N ratio	Chl *a*	VAZ : Chl *a*	De‐epox.
RGR	0.1176 (0.0903)	−**0.5570 (<0.0001)**	−**0.4218 (<0.0001)**	−**0.2547 (0.0011)**	0.0337 (0.6668)	**0.2013 (0.0082)**	−**0.2911 (0.0001)**	−0.1269 (0.0979)
*F_v_*/*F_m_*		−0.1117 (0.1436)	0.0943 (0.2293)	**0.3068 (<0.0001)**	−**0.3125 (<0.0001)**	−0.1148 (0.1325)	−**0.2828 (0.0002)**	−**0.3954 (<0.0001)**
Mannitol			**0.3691 (<0.0001)**	**0.1895 (0.0154)**	0.0288 (0.7131)	−**0.1785 (0.0191)**	**0.2444 (0.0014)**	0.1035 (0.1769)
Carbon				**0.3053 (<0.0001)**	0.0758 (0.3327)	−0.0912 (0.2436)	0.1186 (0.1323)	−**0.1964 (0.0127)**
Nitrogen					−**0.6189 (<0.0001)**	−0.1418 (0.0700)	−0.0494 (0.5309)	−**0.2317 (0.0033)**
C : N ratio						0.0779 (0.3194)	**0.1928 (0.0144)**	**0.1993 (0.0114)**
Chl *a*							−**0.3431 (<0.0001)**	−0.0841 (0.2729)
VAZ : Chl *a*								**0.3372 (<.0001)**

Notes

*n* = 80, except *n* = 96 for the correlation of RGR and F_v_/F_m_ due to the inclusion of Spitsbergen material. Outliers (see Section 2.2.6) were not included in the analysis and further reduced *n* in the following comparisons: *n* = 76 for comparisons involving data from C:N analysis; *n* = 79 for comparisons involving xanthophyll pigment data; *n* = 75 for comparisons involving both. Statistically significant values are indicated in bold text.

**Table A5:** Frequency of null alleles per marker and per population of *Laminaria digitata* obtained using FREENA software

	Ld148	Ld158	Ld167	Ld371	Ld531	Ld704	Lo454‐23	Lo454‐24	Lo454‐17	Lo454‐27	Lo454‐28	Lo4‐24
Spitsbergen	0.00001	**0.29297**	**0.07023**	**0.17081**	**0.12798**	0.00001	0	**0.07593**	0.001	0.00001	0	0.00836
Tromsø	0	0.001	0	**0.23131**	0.00001	0	0.02599	0	0.00004	**0.10953**	0.00006	0.00001
Bodø	**0.11562**	0.00001	0.00001	**0.13494**	0.00001	0	**0.20983**	**0.11409**	0.00006	0.00001	0.00006	0.00001
Helgoland	0.00012	0	0	0	**0.12754**	0.01587	**0.09471**	0.00007	0.001	0.001	0.00007	0.001
Roscoff	0.01255	**0.17234**	0.04511	0.01372	0.01204	0.02449	0.01144	**0.072**	0	0.001	0.03801	**0.11747**
Quiberon	0.00002	**0.13067**	0	0.00001	0.00001	**0.06379**	0.00001	0.01969	0.01643	0.001	0.00001	**0.13145**

Notes

Significant values (>0.05) are highlighted in bold text.

**Table A6:** *p*‐values for linkage disequilibrium based on 7,920 permutations using FSTAT for each pair of markers and for each tested population of *Laminaria digitata*.

	Spitsbergen	Tromsø	Bodø	Helgoland	Roscoff	Quiberon	All
Ld148 × Ld158	0.0601	NA	0.03169	0.72551	0.86768	0.24407	0.12702
Ld148 × Ld167	0.48548	0.22184	0.42841	0.39836	0.85303	0.08611	0.23763
Ld148 × Ld371	0.86351	0.67891	0.67071	0.97235	1.000	0.58561	0.95379
Ld148 × Ld531	0.25341	0.92727	0.03485	0.53889	0.86793	0.64343	0.53876
Ld148 × Ld704	0.63838	0.74798	0.42247	0.73977	0.99242	0.95783	0.99255
Ld148 × Lo454‐23	0.57816	0.22096	0.30152	0.04836	0.46035	0.68763	0.15758
Ld148 × Lo454‐24	0.64104	0.73687	0.28207	0.23068	0.92172	0.19432	0.56023
Ld148 × Lo454‐17	NA	0.71098	0.11995	NA	0.67348	0.49886	0.61439
Ld148 × Lo454‐27	0.89369	0.69785	0.36667	NA	NA	NA	0.63232
Ld148 × Lo454‐28	0.22159	0.43359	1.000	1.000	0.68952	0.50316	0.50682
Ld148 × Lo4‐24	0.03573	0.42323	0.42854	NA	0.54268	0.6423	0.32374
Ld158 × Ld167	0.86679	NA	0.76705	0.02525	0.64356	0.12525	0.29356
Ld158 × Ld371	0.15985	NA	0.69912	0.80833	0.52942	0.08106	0.25694
Ld158 × Ld531	0.00328	NA	0.18043	0.14495	0.9149	0.80038	0.05467
Ld158 × Ld704	0.4952	NA	0.42841	0.32866	0.43321	0.54899	0.45783
Ld158 × Lo454‐23	0.02033	NA	0.65568	0.28081	0.50934	0.01995	0.01667
Ld158 × Lo454‐24	0.87917	NA	0.64545	1.000	0.59116	0.01414	0.43258
Ld158 × Lo454‐17	NA	NA	0.06465	NA	0.22841	0.0178	0.00051
Ld158 × Lo454‐27	0.19419	NA	0.71275	NA	NA	NA	0.22614
Ld158 × Lo454‐28	0.19078	NA	1.000	0.20543	0.63182	0.65328	0.3702
Ld158 × Lo4‐24	0.70341	NA	0.87551	NA	0.67866	0.20783	0.62247
Ld167 × Ld371	0.36174	0.00025	1.000	0.34293	1.000	0.3322	0.00455
Ld167 × Ld531	0.05758	0.56717	0.55909	0.04444	0.40821	0.97942	0.13775
Ld167 × Ld704	0.83005	0.60568	0.95101	0.2303	0.99886	0.00379	0.80126
Ld167 × Lo454‐23	0.18662	0.69457	0.38965	0.05707	0.13788	0.26174	0.05972
Ld167 × Lo454‐24	0.30391	0.91503	0.85732	1.000	0.71831	0.03157	0.51402
Ld167 × Lo454‐17	NA	0.69116	0.33434	NA	0.79356	0.16944	0.3524
Ld167 × Lo454‐27	0.92083	0.32462	0.25682	NA	NA	NA	0.58106
Ld167 × Lo454‐28	0.34066	1.000	0.35177	0.49015	0.36124	0.86629	0.43093
Ld167 × Lo4‐24	0.47664	0.32159	0.68194	NA	0.85593	0.09508	0.4077
Ld371 × Ld531	0.76705	0.26389	0.40669	0.72311	0.58157	0.36275	0.47146
Ld371 × Ld704	0.84331	0.36035	0.50631	0.46111	0.11136	0.25328	0.27121
Ld371 × Lo454‐23	0.89356	0.8221	0.68662	0.1923	1.000	0.95732	0.84381
Ld371 × Lo454‐24	0.83624	0.32134	0.31301	0.40543	0.94672	0.04836	0.29495
Ld371 × Lo454‐17	NA	0.31957	0.77841	NA	0.84066	0.1779	0.3404
Ld371 × Lo454‐27	0.35467	0.23144	0.92778	NA	NA	NA	0.45985
Ld371 × Lo454‐28	0.4375	0.57008	0.60114	1.000	0.89609	0.0928	0.37576
Ld371 × Lo4‐24	0.42058	0.2971	0.53636	NA	0.775	0.1327	0.22071
Ld531 × Ld704	0.81288	0.81149	0.12412	0.38674	0.15543	0.61932	0.36818
Ld531 × Lo454‐23	0.07538	0.13788	0.54545	0.84091	0.11351	0.06641	0.03737
Ld531 × Lo454‐24	0.19268	0.92639	0.82361	1,000	0.59975	0.55025	0.81225
Ld531 × Lo454‐17	NA	0.84457	0.15303	NA	0.97071	0.49293	0.71755
Ld531 × Lo454‐27	0.47929	0.31073	0.73043	NA	NA	NA	0.31742
Ld531 × Lo454‐28	0.34861	1.000	1.000	1.000	0.30404	0.90366	0.75455
Ld531 × Lo4‐24	0.52273	0.57109	0.19495	NA	0.12828	0.91831	0.5899
Ld704 × Lo454‐23	0.50972	0.00947	0.53687	0.19646	0.44167	0.7471	0.13674
Ld704 × Lo454‐24	0.4298	0.14419	0.69066	1.000	0.72083	0.80783	0.59609
Ld704 × Lo454‐17	NA	0.68649	0.74924	NA	1.000	0.67487	0.92891
Ld704 × Lo454‐27	0.11439	0.72702	0.63068	NA	NA	NA	0.41111
Ld704 × Lo454‐28	0.24886	1.000	0.52702	1,000	0.44937	0.15909	0.32626
Ld704 × Lo4‐24	0.70265	0.45909	0.84861	NA	1.000	0.67361	0.88182
Lo454‐23 × Lo454‐24	0.92702	0.65505	0.90543	0.65492	0.51919	0.31389	0.86705
Lo454‐23 × Lo454‐17	NA	0.4721	0.34811	NA	0.83005	0.6101	0.70707
Lo454‐23 × Lo454‐27	0.47083	0.65593	0.36629	NA	NA	NA	0.54091
Lo454‐23 × Lo454‐28	0.69482	0.72866	0.34167	0.06098	0.74419	0.9197	0.76465
Lo454‐23 × Lo4‐24	0.35202	0.8721	0.85316	NA	0.55833	0.36427	0.81338
Lo454‐24 × Lo454‐17	NA	0.21427	1.000	NA	0.96174	0.09684	0.32399
Lo454‐24 × Lo454‐27	0.23636	0.75164	1.000	NA	NA	NA	0.82412
Lo454‐24 × Lo454‐28	1,000	1,000	0.21465	1.000	0.89381	0.88232	0.96944
Lo454‐24 × Lo4‐24	0.11982	0.37437	0.07917	NA	0.40707	0.09482	0.02134
Lo454‐17 × Lo454‐27	NA	0.72412	1.000	NA	NA	NA	0.77513
Lo454‐17 × Lo454‐28	NA	1.000	1.000	NA	0.23914	0.63902	0.61717
Lo454‐17 × Lo4‐24	NA	0.6721	1.000	NA	0.1197	0.00745	0.05101
Lo454‐27 × Lo454‐28	0.02109	0.06742	1.000	NA	NA	NA	0.00303
Lo454‐27 × Lo4‐24	0.86869	0.84432	1.000	NA	NA	NA	0.98295
Lo454‐28 × Lo4‐24	0.51023	1.000	0.50189	NA	1.000	0.41717	0.70063

Notes

Microsatellite loci published for *Laminaria digitata* (Ld; Billot et al., 1998) and *Laminaria ochroleuca* (Lo; Coelho et al., 2014). The *p*‐value after multiple testing correction for 5% nominal level is 0.000126. No linkage disequilibrium is significant in the dataset.

**Table A7:** Estimates of genetic diversity and deviation from random mating for each locus and each population of *Laminaria digitata* tested in this study.

Population	Locus	*n*	N_a_	AR	P_a_	H_e_	H_o_	F_IS_
Spitsbergen	Ld148	26	3	2.808	0	0.212	0.231	−0.110
	Ld158	25	4	4	1	0.709	0.200	0.712
	Ld167	26	5	4.802	1	0.714	0.615	0.121
	Ld371	26	9	8.355	0	0.796	0.500	0.360
	Ld531	26	3	2.966	1	0.520	0.308	0.396
	Ld704	21	2	2	0	0.136	0.143	−0.077
	Lo454‐23	26	5	4.61	0	0.531	0.615	−0.182
	Lo454‐24	26	5	4.581	0	0.281	0.231	0.164
	Lo454‐17	26	1	1	0	0.000	0.000	#NV
	Lo454‐27	26	2	2	0	0.510	0.538	−0.077
	Lo454‐28	26	3	2.808	0	0.446	0.500	−0.144
	Lo4‐24	26	2	2	0	0.483	0.462	0.025
Tromsø	Ld148	30	3	3	0	0.603	0.800	−0.350
	Ld158	30	1	1	0	0.000	0.000	#NV
	Ld167	30	4	3.883	1	0.274	0.300	−0.113
	Ld371	30	9	8.456	1	0.793	0.367	0.530
	Ld531	30	3	2.89	0	0.159	0.167	−0.068
	Ld704	30	4	3.89	0	0.393	0.467	−0.209
	Lo454‐23	30	5	4.614	0	0.667	0.600	0.086
	Lo454‐24	30	4	3.976	0	0.444	0.500	−0.146
	Lo454‐17	30	2	2	0	0.398	0.400	−0.023
	Lo454‐27	30	2	1.976	0	0.097	0.033	0.649
	Lo454‐28	30	2	1.7	1	0.033	0.033	−0.017
	Lo4‐24	30	4	3.975	0	0.569	0.533	0.047
Bodø	Ld148	32	4	3.963	1	0.634	0.438	0.299
	Ld158	32	3	2.882	0	0.203	0.188	0.061
	Ld167	31	8	7.664	2	0.839	0.839	−0.016
	Ld371	31	15	12.946	1	0.871	0.613	0.285
	Ld531	32	3	2.874	0	0.177	0.156	0.101
	Ld704	32	5	4.884	1	0.684	0.688	−0.021
	Lo454‐23	32	6	5.538	0	0.635	0.313	0.500
	Lo454‐24	32	5	4.542	1	0.590	0.438	0.246
	Lo454‐17	32	2	1.656	0	0.031	0.031	−0.016
	Lo454‐27	32	2	1.999	0	0.173	0.188	−0.103
	Lo454‐28	32	2	1.656	0	0.031	0.031	−0.016
	Lo4‐24	32	3	2.963	0	0.549	0.594	−0.098
Helgoland	Ld148	34	2	2	0	0.504	0.500	−0.008
	Ld158	35	3	2.6	1	0.520	0.600	−0.169
	Ld167	35	4	3.687	0	0.512	0.600	−0.189
	Ld371	35	9	7.861	0	0.618	0.657	−0.078
	Ld531	35	4	3.937	0	0.493	0.314	0.353
	Ld704	35	2	2	0	0.487	0.457	0.048
	Lo454‐23	35	3	2.843	0	0.535	0.371	0.295
	Lo454‐24	35	2	1.6	0	0.029	0.029	−0.014
	Lo454‐17	35	1	1	0	0.000	0.000	#NV
	Lo454‐27	35	1	1	0	0.000	0.000	#NV
	Lo454‐28	35	2	1.6	0	0.029	0.029	−0.014
	Lo4‐24	35	1	1	0	0.000	0.000	#NV
Roscoff	Ld148	28	7	6.678	2	0.782	0.750	0.023
	Ld158	28	4	3.749	1	0.477	0.250	0.466
	Ld167	28	7	6.718	2	0.727	0.643	0.100
	Ld371	28	15	14.025	0	0.906	0.893	−0.003
	Ld531	28	4	3.997	0	0.658	0.571	0.115
	Ld704	28	4	3.691	0	0.475	0.429	0.081
	Lo454‐23	28	9	8.211	1	0.779	0.714	0.067
	Lo454‐24	28	4	3.737	1	0.410	0.286	0.290
	Lo454‐17	28	2	2	0	0.299	0.357	−0.217
	Lo454‐27	28	1	1	0	0.000	0.000	#NV
	Lo454‐28	28	2	2	0	0.249	0.214	0.125
	Lo4‐24	28	3	2.691	0	0.105	0.036	0.652
Quiberon	Ld148	28	6	5.497	0	0.664	0.643	0.014
	Ld158	28	3	2.941	0	0.257	0.143	0.434
	Ld167	28	5	4.441	0	0.318	0.321	−0.031
	Ld371	28	11	9.927	1	0.836	0.786	0.043
	Ld531	28	4	3.737	0	0.497	0.500	−0.023
	Ld704	28	3	2.75	0	0.408	0.321	0.199
	Lo454‐23	28	7	6.191	1	0.519	0.536	−0.050
	Lo454‐24	28	6	5	1	0.344	0.321	0.047
	Lo454‐17	28	4	3.999	1	0.603	0.607	−0.025
	Lo454‐27	28	1	1	0	0.000	0.000	#NV
	Lo454‐28	28	2	2	0	0.503	0.536	−0.084
	Lo4‐24	28	3	2.75	0	0.328	0.179	0.446

Notes

Locus, microsatellite loci published for *Laminaria digitata* (Ld; Billot et al., 1998) and *Laminaria ochroleuca* (*L*_o_; Coelho et al., 2014); *n*, number of individuals for which the marker amplified; N_a_, number of observed alleles; AR, allelic richness standardized for equal sample size (21 individuals); P_a_, number of private alleles per locus; H_e_, expected heterozygosity; H_o_, observed heterozygosity; F_IS_, fixation index (inbreeding coefficient) of individuals with respect to local subpopulation; #NV, no calculation of F_IS_ in monomorphic loci. Note that in Helgoland, Roscoff and Quiberon, the locus Lo454‐27 is fixed while it is polymorphic for Spitsbergen, Tromsø and Bodø. This explains why this locus was not included in the study of Robuchon et al. (2014).

**Table A8:** Fixation index (F_ST_) for each pair of the *Laminaria digitata* populations tested in this study

	Tromsø	Bodø	Helgoland	Roscoff	Quiberon
Spitsbergen	0.422	0.344	0.468	0.338	0.444
Tromsø		0.293	0.582	0.466	0.520
Bodø			0.442	0.319	0.385
Helgoland				0.156	0.352
Roscoff					0.162

Notes

All *p*‐values obtained with 300 permutations using FSTAT were 0.003 and therefore significant (the *p*‐value corrected for multiple testing is .003).
